# 3D‐Printing inside the Glovebox: A Versatile Tool for Inert‐Gas Chemistry Combined with Spectroscopy

**DOI:** 10.1002/hlca.201500502

**Published:** 2016-04-22

**Authors:** Felix Lederle, Christian Kaldun, Jan C. Namyslo, Eike G. Hübner

**Affiliations:** ^1^ Institute of Organic ChemistryClausthal University of TechnologyLeibnizstr. 6DE‐38678Clausthal‐Zellerfeld

**Keywords:** 3D‐Printing, Online analytics, Inert‐gas synthesis, Trimethylaluminium, NMR Spectroscopy

## Abstract

3D‐Printing with the well‐established ‘Fused Deposition Modeling’ technology was used to print totally gas‐tight reaction vessels, combined with printed cuvettes, inside the inert‐gas atmosphere of a glovebox. During pauses of the print, the reaction flasks out of acrylonitrile butadiene styrene were filled with various reactants. After the basic test reactions to proof the oxygen tightness and investigations of the influence of printing within an inert‐gas atmosphere, scope and limitations of the method are presented by syntheses of new compounds with highly reactive reagents, such as trimethylaluminium, and reaction monitoring *via* UV/VIS, IR, and NMR spectroscopy. The applicable temperature range, the choice of solvents, the reaction times, and the analytical methods have been investigated in detail. A set of reaction flasks is presented, which allow routine inert‐gas syntheses and combined spectroscopy without modifications of the glovebox, the 3D‐printer, or the spectrometers. Overall, this demonstrates the potential of 3D‐printed reaction cuvettes to become a complementary standard method in inert‐gas chemistry.

## Introduction

3D‐Printing has found its way into daily life and evolved from more experimental and specialized setups towards the mass market and is used for efficient production setups. Besides spectacular demonstrations of 3D‐printing (ranging from food to concrete), the main impact of 3D‐printers on our daily life probably is their rapidly increasing dissemination (beginning with the availability in market stores up to the use in small‐scale productions) nowadays 1, 2. Most notably, printers based on ‘Fused Deposition Modeling’ (FDM), which is realized by building up the desired objects layer by layer of molten polymer, have arisen as most cost‐efficient and fast technology. They are available in the price range around $1000. Primarily, acrylonitrile butadiene styrene (ABS) and polylactide (PLA) are used as printing material, of which ABS shows excellent mechanical properties. The use of various other polymers, such as polyethylene terephthalate (PET), polyamides, and the chemically inert polypropylene (PP) as well as silicones is increasing, although more complex printing setups may be necessary, as well as a reduced mechanical stability may be achieved. Consequently, 3D‐printing has successfully been applied for the construction of chemical reactions vessels. For example, microreactors printed out of silicone allowed to distinguish between two different products yielded from the same starting materials by the geometry of the printed reactor 3, 4. Closely packed tubes printed out of PP were used for the hydrothermal syntheses of metal–organic frameworks 5, 6. The thin channels in a matrix of PP or PET were used as flow reactors for the synthesis and further analytical applications of different nanoparticles 7, 8, 9. A combination of PP and silicones was used to print ‘reaction cubes’ in which multistep reactions were realized by overturning the appropriate sides of the cube to mix the desired starting materials step‐by‐step, while catalysts were embedded into the silicon matrix 10.

**Figure 1:**
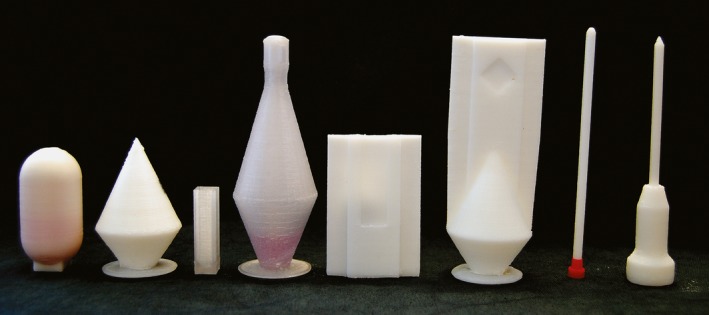
3D‐Printed reaction flasks, cuvettes, and objects. From left: round‐bottom flask made of ABS‐N (F1), conical flask made of ABS‐N (F2), UV cuvette made of ABS‐K (F3), inert‐gas flask with UV cuvette made of ABS‐K (F4), IR cuvette made of ABS‐N (F5), inert‐gas flask with IR cuvette made of ABS‐N (F6), NMR tube made of ABS‐N (F7), and inert‐gas flask/spinner with NMR tube made of ABS‐N (F8).

The concept for inert‐gas chemistry within 3D‐printed reaction vessels and cuvettes presented here is based on a commercially available 3D‐printer without any modifications. The 3D‐printer is ready for operation just after insertion into a glovebox, which is not modified either. The set of reaction flasks presented here (*Fig.* 1) allows for inert‐gas chemistry at elevated temperatures by maintaining the nitrogen atmosphere after removal from the glovebox. Accompanying measurements of UV/VIS, IR, and NMR spectra are possible without opening the reaction flasks. Additionally, after completion of the reaction, pure products can easily be isolated without contamination from the ABS matrix material.

## Results and Discussion

For all prints, an appropriate sized FDM‐3D‐printer was used, which was inserted into the glovebox *via* the vacuum chamber over night. To communicate with the printer inside the glovebox, the USB signal was transmitted *via* ethernet and the ethernet signal was passed into the glovebox *via* PowerLAN using the existing power supply of the glovebox, so no additional wiring was necessary. After insertion into the inert‐gas atmosphere, the general aspects of printing inside an oxygen‐free atmosphere were investigated. The polymer used for all prints was ABS due to its extraordinary mechanical stability. In general, ABS is a graft copolymer of styrene and acrylonitrile (SAN) on polybutadiene. As a result of phase separation (phases of different refractive indices) it is usually turbid 11. With the help of ‘contrast matching’ *via* the addition of acrylates, this effect may be avoided and a clear polymer is obtained. Consequently, such a polymer (ABS‐K) was used to print the UV/VIS cuvettes. All other flasks were printed out of an unmodified ABS (ABS‐N) to keep the number of reactive residues within the matrix polymer as low as possible. Additionally, the C=O band of the acrylates would block a significant region of the IR spectrum and ABS‐N was the choice for IR cuvettes, too. The glass transition temperature (*T*_g_) of printed stripes of both polymers was determined *via* differential scanning calorimetry to 98 °C (ABS‐K) and 111 °C (ABS‐N) and therefore the temperature range for heating up the printed reaction vessels is limited to roughly 100 °C. Samples of both polymers were dissolved in acetone, the soluble part isolated and the molecular weight of the matrix material determined to *M*_n_ = 55,000 g/mol (ABS‐K) with a molecular weight distribution of 2.08 and *M*_n_ = 67,000 g/mol (ABS‐N) with a molecular weight distribution of 2.25. The presence of C=O groups was checked *via* ATR‐IR and they were found at 1730 cm^−1^ in case of ABS‐K, while no absorption in this region was noticeable for ABS‐N. Finally, a set of printed stripes was analyzed *via Karl–Fischer* titration with regard to the total residual water content. The water content of both polymers after printing was found to be around 200 ppm (0.028 ± 0.004% for ABS‐K and 0.024 ± 0.005% for ABS‐N) and therefore the amount of water released from the inner surface of printed reaction vessels can be considered to be extremely low and does not interfere with the use of highly reactive reagents.

**Figure 2:**
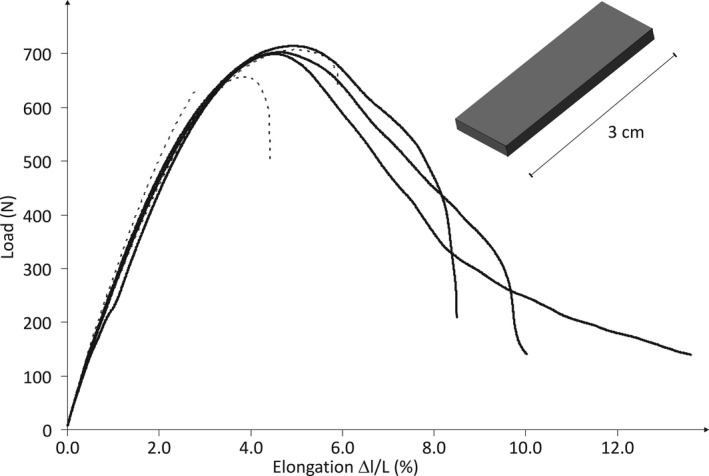
Load–strain curves of plates made of ABS‐N printed within a N_2_ atmosphere (solid line) and at air (dashed line). Elastic modulus: *E*(air) = 0.87 ± 0.03 kN/mm^2^, *E*(N_2_) = 0.85 ± 0.04 kN/mm^2^; tensile strength: *σ*_M_(air) = 19.4 ± 0.8 N/mm^2^, *σ*_M_(N_2_) = 21.6 ± 0.2 N/mm^2^.

Tensile tests have been performed to show the influence of printing within an inert‐gas atmosphere. Printed plates made of ABS‐N showed a significant increase in the elongation at break from *ε*_B_(air) = 4.4 ± 1.5% to *ε*_B_(N_2_) = 10.7 ± 2.6% which is an indication for a significantly improved layer adhesion if printed within an inert‐gas atmosphere. This is an interesting result for 3D‐printing in general, and furthermore, an important aspect for the impermeability of printed reactions flasks (*Fig.* 2).

To get an idea of the impermeability of the printed flasks against oxygen and H_2_O, the intrusion was roughly estimated by the surface area (200 cm^2^), the wall thickness (3 mm), and the permeability of ABS against oxygen (approx. 80 ml mm/m^2^/day/atm O_2_) and water (approx. 8 g mm/m^2^/day H_2_O) to 5 μmol O_2_/day and 3 μmol H_2_O/day 11. Although imperfect printed walls and imperfect layer adhesion are neglected in this estimation, it is a promising result for gas‐tight reaction flasks. For first tests of the leak tightness, the flasks F1 and F2 (*Fig.* 1) were filled with water and isooctane (containing rhodamine and rubrene, respectively, as dyes) during short pauses of the print. The flasks were cooled down to −40 °C and heated up to 95 °C for 24 h subsequently, and no weight loss (indicating a leakage) was noticeable. Since the printing of the round upper shell of the flask F1 was found to be challenging, all following experiments have been performed in flask F2, whose conical closing can be printed precisely and reproducibly. The dissolution properties of ABS allow for the usage of strong polar solvents, such as water and lower alcohols, as well as strong nonpolar solvents, such as *n*‐alkanes. To check the possibility of measuring accurate spectra directly in printed reaction flasks, UV/VIS, IR, and NMR spectra of pure substances have been measured before setting up reactions inside the glovebox. All cuvettes have been dimensioned according to their glass or metal counterparts and directly fit into standard spectrometers (*Fig.* 1).

**Figure 3:**
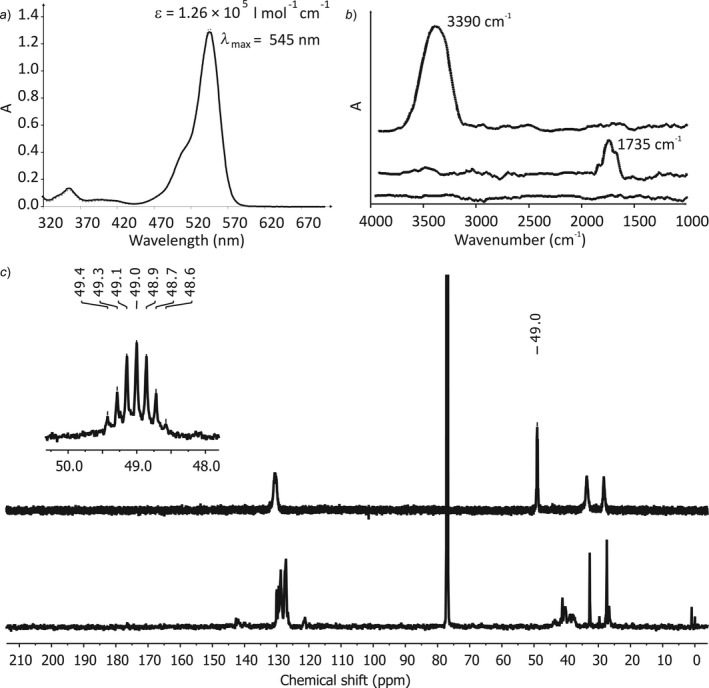
Spectroscopy in 3D‐printes cuvettes. *a*) UV/VIS Spectra of rhodamine B (0.01 mm) in MeOH (*vs*. MeOH background) in glass cuvettes (dashed line) and cuvettes F3 (solid line). *b*) IR Spectra of pentane (bottom), 5 wt% acetone in pentane (middle), and 5 wt% EtOH in pentane (top) (*vs*. pentane background in all cases) in cuvette F5. *c*) ^13^C‐NMR Spectrum of ABS‐N in CDCl_3_ (bottom) and CD_3_OD in NMR tube F7.

The UV/VIS spectrum of the empty cuvette F3 made of ABS‐K (*vs*. air) shows a strongly increasing absorption above 350 nm and consequently UV/VIS spectra can be measured in the range from 900 – 350 nm. The measurement of a rhodamine B solution in the printed cuvette F3 almost perfectly matches the spectrum measured in the quartz glass cuvette (*Fig.* 3 *a*) and the extinction coefficient was determined to *ε *= 1.26 × 10^5^ l/mol/cm at *λ*_max_ = 545 nm, which is close to the specifications of the manufacturer. The excitation–emission matrix of a plate of ABS‐K shows a weak fluorescence beginning at an excitation wavelength of *λ*_exc_ **= 410 nm with a maximum at *λ*_exc_ = 380 nm. Consequently, the emission maximum of a 0.001 mm solution of rhodamine B in water was correctly determined to *λ*_em_ = 579 nm (*λ*_exc_ = 545 nm) in absolute accordance with the measurement in a quartz glass cuvette. The detected fluorescence intensity was lowered by a factor of 5 compared to the glass cuvette. The ATR‐IR spectra could be recorded in the range from 1500 – 2800 cm^−1^ and > 3100 cm^−1^ using cuvette F5 printed out of ABS‐N, which is in accordance with the absorption spectrum of the pure polymer (*Fig.* 3 *b*). The C=O band of acetone in nonpolar pentane was determined to $\tilde{v}$ = 1735 cm^−1^ which is a hypsochromic shift compared to the measurement of the pure substance ($\tilde{v}$ = 1712 cm^−1^) and is in good accordance with results reported in the literature for diluted solutions in nonpolar solvents 12. The OH‐stretching vibration of MeOH in pentane was determined to $\tilde{v}$ =* *3390 cm^−1^ in accordance with literature results again, and is dominated by H‐bridges formed at the given concentration 13. Finally, the ^13^C‐NMR spectrum of pure ABS‐N dissolved in CDCl_3_ shows only comparably weak and narrow signals close to 130 ppm (primarily aromatic carbons) and at 25 – 45 ppm (aliphatic carbons). Consequently, it was possible to record a very neat spectrum of CD_3_OD in the printed NMR tube F7 and the ^1^ *J*_C,D_ coupling constant was correctly determined to 20 Hz (*Fig.* 3 *c*) 14.

**Figure 4:**
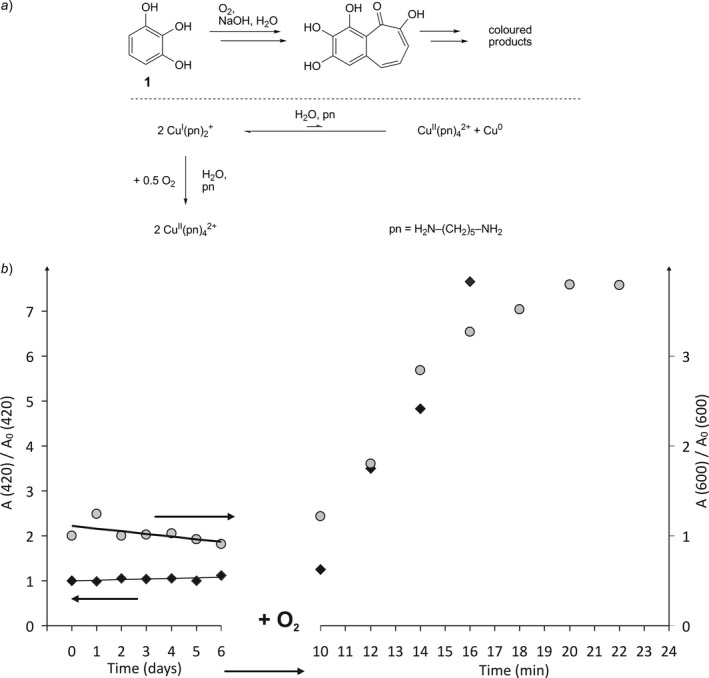
Inert‐gas chemistry combined with UV/VIS spectroscopy performed in reaction cuvette F4. *a*) Top: Test Reaction of pyrogallol with oxygen under basic conditions. Bottom: Disproportionation of copper(I) in aqueous solution in the presence of the non‐chelating ligand 1,5‐diaminopentane and oxidation of copper(I) to copper(II) in the presence of oxygen. *b*) Plot of the absorption at 420 nm *vs*. initial absorption at 420 nm of the pyrogallol solution (black) and plot of the absorption at 600 nm *vs*. initial absorption at 600 nm of the CuCl reaction mixture (gray). Solid lines are a guide for the eye.

After the general measurements of spectra have proven to be possible, the actual impermeability against oxygen was checked *via* an UV spectrometric test reaction for oxygen. The reaction flask F4 with an attached and printed UV cuvette was printed within the glovebox and filled with an approx. 0.15m solution of pyrogallol (**1**) in 0.65m aqueous NaOH during a short pause. After finishing the print, flask F4 was discharged out of the glovebox and UV/VIS spectra were recorded on a daily basis. In a basic solution, pyrogallol decomposes extremely fast into intensively colored reaction products in the presence of oxygen. Therefore, it is well‐known and used as a quantitative and qualitative test reaction for oxygen (*Fig.* 4 *a*) 15, 16.

The absorption at 420 nm remains unchanged during 6 days (*Fig.* 4 *b*), and consequently, flask F4 shows an impressive impermeability against oxygen. After puncturing the flask to allow air to insert *via* a small hole, an intense brown color of the solution was observed within few minutes which was reflected in the fast increase in the absorption at 420 nm.

**Scheme 1:**
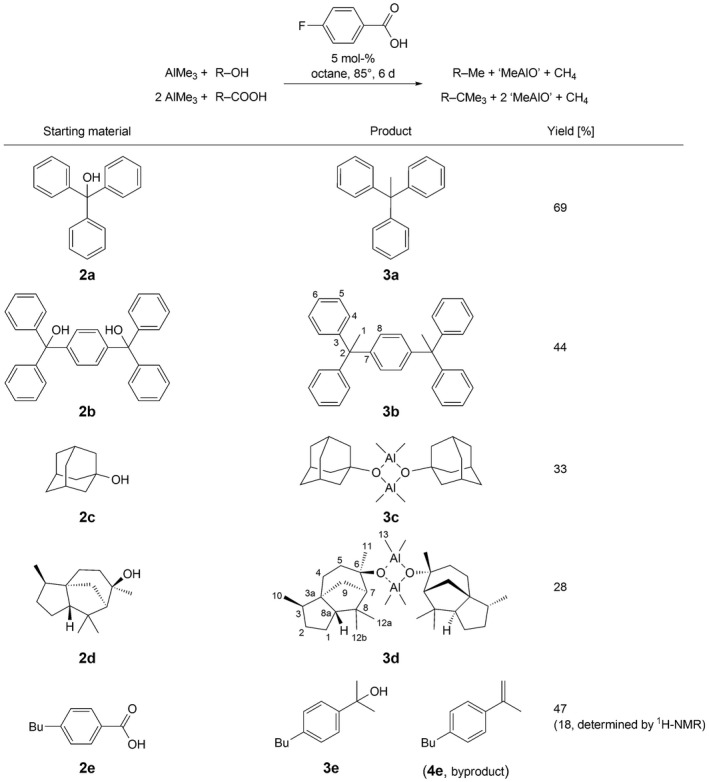
Reaction products obtained by reduction of alcohols and carboxylic acids with trimethylaluminium in flasks F2 and F6.

After the reaction flask has proven to be sufficiently oxygen tight, it was used to monitor the equilibrium between copper(I) and copper(II). This allows to check the usability for inorganic aqueous reactions and is an additional proof for the impermeability. In the presence of non‐chelating ligands, the equilibrium between copper(I) and copper(II) is located strongly on the side of copper(I) (~3 mol‐% Cu(II) in the presence of 1,5‐diaminopentane) (*Fig.* 4 *a*) 17, 18. Reaction flask F4 was filled with CuCl and a solution of 1,5‐diaminopentane in H_2_O during two short pauses of the print and UV/VIS spectra were recorded on a daily basis (*Fig.* 4 *b*). A slight indication for a disproportionation at the first day was noticeable by a slight increase in the characteristic absorption at 600 nm and by the visible formation of tiny amounts of elemental copper. During the following days, equilibration (probably by reaction of the copper(II) with dispersed elemental copper) took place and the absorption at 600 nm remained unchanged. After puncturing the flask, an immediate occurrence of a deep blue color indicated the irreversible formation of copper(II) within a few minutes (*Fig.* 4 *b*). Based on these experiments, which demonstrated the UV/VIS spectroscopic opportunities and inertness of the flasks against various reagents, the synthetic potential of printed reaction vessels in the presence of highly reactive starting materials was evaluated. Consequently, the reductive methylation of alcohols and carboxylic acids by trimethylaluminum (AlMe_3_) was investigated (*Scheme* 1) 19, 20.

In contrast to literature, octane was chosen as solvent and therefore 4‐fluorobenzoic acid instead of benzoic acid was used as catalyst, as it shows a good solubility in octane, the F–aryl bond tolerates AlMe_3_ and only comparably low‐boiling products are formed out of the catalyst 21. The known reduction of triphenylmethanol (**2a**) was performed in the reaction cuvette F6, which was filled with the starting material, the catalyst, and a solution of AlMe_3_ in octane during two short pauses. After discharging from the glovebox, the flask was heated to 85 °C for 6 days, and after opening the reaction vessel (and aqueous workup), pure triphenylethane (**3a**) was received in 69% yield. Based on this promising result, the reduction of the aromatic alcohol **2b** under analogous conditions in the reaction flask F2 was tested and yielded the neat reduction product **3b** as new and fully characterized compound. Owed to the limited reaction temperature of about 100 °C, a reduction of the tertiary aliphatic alcohols **2c** and **2d** could not be observed. Instead, the reaction of (+)‐cedrol (**2d**) with AlMe_3_ using the flask F2 and aqueous workup afforded an air‐stable compound, which showed a significant shift of the **C**–O C‐atom from *δ *= 75.2 – 81.8 ppm in the ^13^C‐NMR spectrum compared to the starting material. While it was not possible to detect a signal for aluminum *via* ^27^Al‐NMR after acid digestion with H_2_SO_4_/H_2_O_2_, the aluminum content could be determined *via* ICP‐OES to 10.19 wt%. This is in accordance with the dimeric dimethylaluminum compound **3d**. The characteristic strong Al–C vibrational band was detected at 690 cm^−1^ in the ATR‐IR spectrum 22 and the Al–C**H**_3_ Me protons were detected at −2.9 ppm ((D_8_)toluene, 70 °C) in the ^1^H‐NMR spectra as a unique singlet. High‐resolution mass spectra doubtlessly confirmed the structure of **3d** by the mass peak of [*M* – Me]^+^, which is typical for compounds of the type [Me_2_Al‐(*μ*‐OR)]_2_ 23. Furthermore, the known dimethylaluminum compound **3c** was prepared for comparison and has been characterized as the assumed dimer *via* mass spectrometry 20. The extraordinary stability of the new enantiopure dimethylaluminum compound **3d** is worth to be pointed out, since the Al–CH_3_ Me groups not only withstand the aqueous workup and subsequent atmospheric water and oxygen for a longer period of time, but also treatment with semiconcentrated HCl. The dimethylaluminum species **3d** showed to be significantly more stable than the comparable compound **3c**. Consequently, it is currently under investigation as Me transfer agent toward transition metals.

**Scheme 2:**
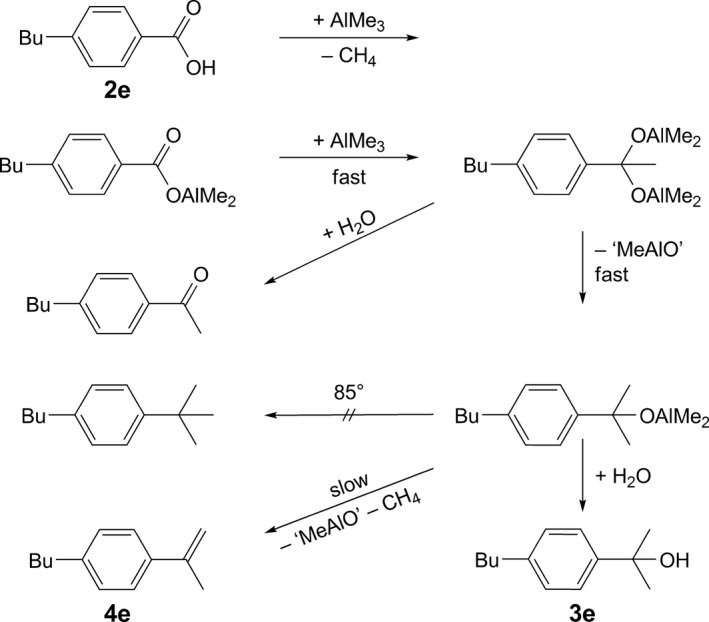
Stepwise reduction of the carboxylic acid **2e** with AlMe_3_ and reaction mechanism discussed on the basis of isolated products and IR spectroscopic measurements in reaction flask F6.

The printed reaction cuvette F6 offers the opportunity to monitor the reduction process with highly reactive AlMe_3_ *via* IR spectroscopy without the need to open the flask. Obviously, the reduction of the carboxylic acid **2e** serves as a convenient experimental system to be examined *via* the C=O vibrational band. The reduction of the carboxylic acid to the corresponding *tert*‐butyl group (*δ* ^t^Bu = 1.41 ppm), expected according to literature, could not be observed at the comparably low reaction temperature of 85 °C. Furthermore, the formation of the acetophenone derivative (*δ*_Me_ = 2.49 ppm) could be excluded according to the ^1^H‐NMR spectra of the isolated reaction products after opening the flask (*Scheme* 1) 19, 24, 25, 26, 27. Instead, the tertiary alcohol **3e** was isolated as main product; consequently lowering the process temperature to 85 °C can be considered as a convenient method to reduce carboxylic acids to the corresponding tertiary alcohol. Due to the long reaction time of 6 days (which has not been optimized to increase the yield of alcohol), a slow elimination to yield the alkene **4e** was noticeable. The main interesting aspect of the reduction process was the fast disappearance of the C=O vibrational band in the IR spectra, which was nearly undetectable directly after discharging flask F6 from the glovebox and before heating up the reaction mixture. All together, this leads to the rather detailed reaction sequence presented in *Scheme* 2: After the instantaneous deprotonation of the carboxylic acid (which is backed by the absence of OH vibrational bands in the IR spectrum measured directly after discharging from the glovebox), a fast transfer of the first Me group can be assumed, which is indicated by the immediate loss of the C=O vibrational band. Since the acetophenone derivative could not be isolated, the second Me group has to be transferred comparably fast, too. This allows the alcohol **3e** to be isolated after aqueous workup. Slowly, the elimination of the aluminum alcoholate to the isolated alkene **4e** takes place, while the low temperature prevents formation of the alkane 28, 29.

**Figure 5:**
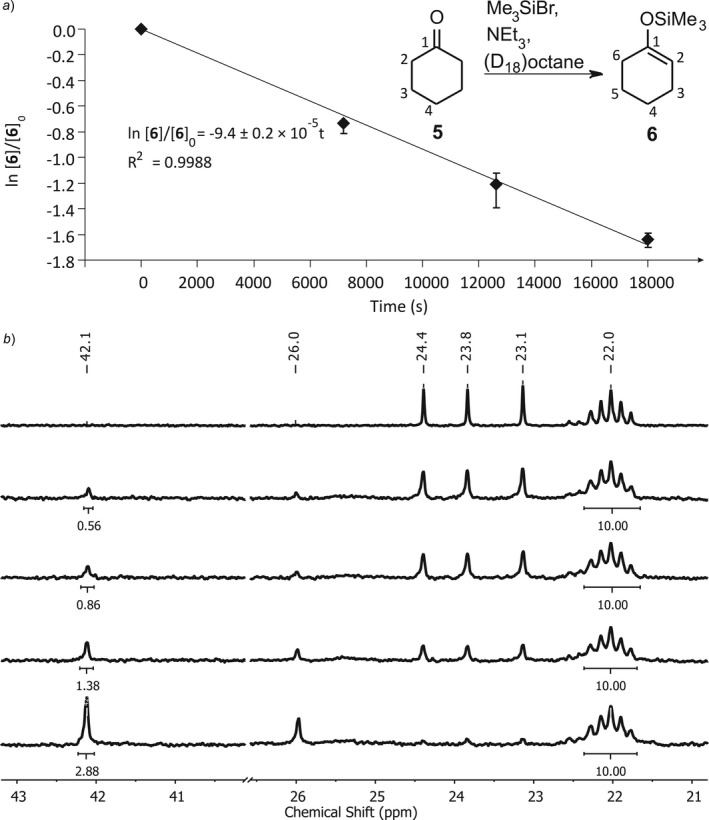
Reaction of cyclohexanone (**5**) with TMSBr. *a*) Determination of the reaction rate constant *k*. Error bars are calculated from uncertainties of the integral values in the NMR spectra. *b*) ^13^C‐NMR Spectra measured in flask F8 directly after printing and discharging the flask from the glovebox (bottom), and after 120, 210, and 300 min as well as control measurement of the isolated reaction solution in a glass NMR tube (top).

After having proven the practicability of printed flasks for monitoring inert‐gas reactions with UV/VIS and IR spectroscopy, NMR spectroscopy was investigated as more challenging analytical technology. To maintain the possibility of larger scaled reactions inside the printed reactions vessels as synthetical approach, the typical spinner of a NMR setup was designed as hollow reaction chamber. The directly attached and printed NMR tube was used for the measurement, while the whole spinner/tube combination fits into an unmodified NMR spectrometer. The reaction flask F8 was used to follow the enolisation of cyclohexanone (**5**) toward the silyl ether **6** (*Fig.* 5) by ^13^C‐ and ^29^Si‐NMR spectroscopy 30, 31.

F8 was filled with a solution of approx. 0.1 g of the starting material **5** and trimethylsilyl chloride (TMSCl) or bromide (TMSBr), respectively, in (D_18_)octane as a solvent and subsequently with triethylamine during two short pauses of the print. After discharging flask F8 from the glovebox and the measurement of initial NMR spectra, the flask was heated in an aluminum bead bath (to prevent contamination of the flask with silicon oil) to 85 °C (internal temperature 70 °C) and NMR spectra were recorded on a regular basis. With TMSCl, no indication of enolisation was noticeable after several days of reaction time, which is in accordance with the comparably low reactivity 31. In contrast, by treatment of **5** with TMSBr, which is more reactive by a factor of ~10^5^, after a few hours at 70 °C, the characteristic olefinic signals of **6** at *δ *= 150.9 (C(1)) and 103.4 ppm (C(2)) as well as the set of signals of the aliphatic C‐atoms at *δ *= 24.4 (C(3)), 23.8 (C(4)) and 23.1 ppm (C(5)) could clearly be detected in the ^13^C‐NMR spectra. The consumption of the starting material **5** was monitored *via* the decrease in the signal of the C‐atoms at *δ *= 42.1 (C(2)/C(2′)) and 26.0 ppm (C(4)). Since the silyl ether formation proceeds *via* a rate‐determining pre‐equilibrium of TMSBr, NEt_3_, and the ketone to an intermediate, which decomposes to the enolised product following a first‐order rate law, it was possible to determine the reaction rate constant to *k *=* *9.4 ± 0.2 × 10^−5^ s^−1^ (70 °C, octane) (*Fig.* 5 *a*). Compared to known results in literature, this nicely matches the expected range 31. Additionally, ^29^Si‐NMR spectra were recorded in the silicon‐free NMR tube/spinner combination. With the help of comparative NMR measurements of the treatment of TMSCl with EtOH and H_2_O in (D_12_)cyclohexane, the ^29^Si‐NMR signals were doubtlessly assigned (*δ*_Si_ = 24.3 (Me_3_SiBr), 14.0 (**6**) and 6.8 ppm Me_3_Si–O–SiMe_3_). The NMR spectra of the reaction mixture in flask F8 revealed a minor amount of hydrolysis (~15 mol % of the initial TMSBr) after 300 min parallel to the product formation. After total consumption of the starting material **5** was achieved, flask F8 was opened and a control measurement recorded in a conventional NMR tube (*Fig.* 5 *b*).

**Figure 6:**
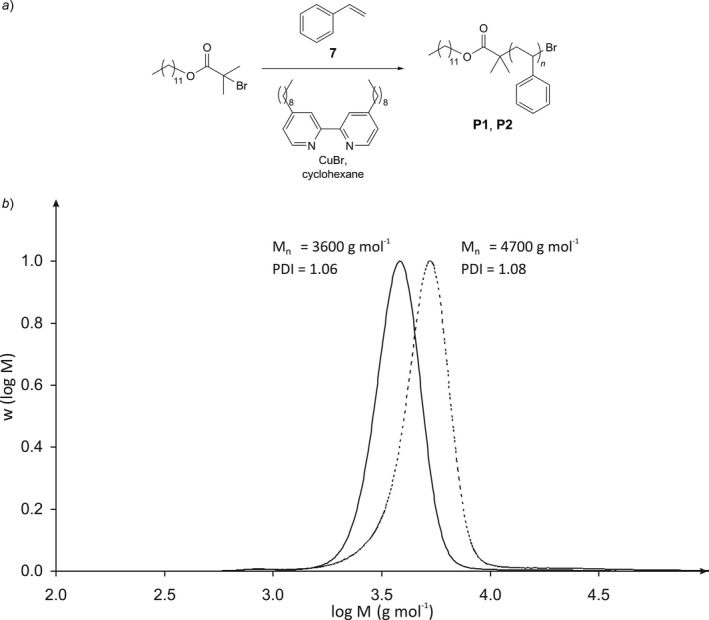
ATRP of styrene in cyclohexane in flask F2. *a*) Reaction conditions adapted to the nonpolar medium. *b*) Molecular weight distribution achieved. Ratio [cyclohexane]:[styrene]:[ligand]:[Cu(I)]:[initiator]: 1000:100:2:1:0.2. Polymerization time: 21 h (**P1**, solid line) and 56 h (**P2**, dashed line).

After examining the different analytical methods in 3D‐printed reaction cuvettes and their stability against highly reactive reagents, the choice of suitable solvents was finally investigated by the controlled atom transfer radical polymerization (ATRP) of styrene in the reaction flask F2 32, 33. Besides the necessary absence of oxygen, the rather similar solubility of the reaction product, polystyrene (PS), and the reaction flask printed out of ABS is the challenging aspect in this case. (*R*)‐Limonene is known to dissolve PS, but not ABS 34. Nevertheless, it cannot be used as reaction medium in this case, since it is known to polymerize by a radical mechanism itself 35. Fortunately, the structurally related methylcyclohexane and cyclohexane turned out to be suited as solvents as well according to solvation tests of small stripes of ABS‐N at 80 °C. Both solvents are just at the edge to a non‐solvent for polystyrene with an upper critical solution temperature (UCST) of approx. 72 °C (methylcyclohexane) and 35 °C (cyclohexane), respectively 36. To minimize the risk of falling below the theta temperature from the start of the polymerization until precipitation of the polymer, cyclohexane was chosen as solvent and the catalytic system adapted to these highly nonpolar reaction conditions (*Fig.* 6).

Flask F2 was filled with CuBr and the ligand 4,4′‐dinonyl‐2,2′‐bipyridine during a first and a solution of styrene and the initiator dodecyl 2‐bromisobutyrate in cyclohexane during a second pause. Flask F2 was heated to 70 °C (internal temperature) for 21 and 56 h, respectively, after discharging from the glovebox. Subsequently, the reaction vessel was opened and without allowing it to cool down, the dark red solution was poured into MeOH and the polymer precipitated. The ATRP proceeded well under the nonpolar reaction conditions and an excellently narrow molecular weight distribution was achieved (*Fig.* 6 *b*), while with increasing reaction time the molar mass of the polystyrene increased as well. The size‐exclusion chromatography traces did not show the slightest indication of impurities dissolved from the reaction flasks. The molar masses achieved are rather low, as expected, although the reaction times chosen were not too short. This correlates with a slow polymerization process and can be explained by the fact of a polymerization in a solution containing only approx. 10 mol‐% of monomer.

These experiments illustrate the limit of suitable solvents for flasks and cuvettes printed out of the ABS matrix. Additionally, they demonstrate the impermeability of the reactions flasks at higher temperatures, hence the diffusion of just ~0.1 mmol O_2_ into the flask would have been sufficient to oxidize the total amount of Cu(I) to Cu(II), and terminate the polymerization.

## Conclusions

In conclusion, 3D‐printing within an inert‐gas atmosphere was investigated and a set of gas‐tight reaction flasks and cuvettes were designed, which allow to perform chemical reactions under inert‐gas atmosphere with highly reactive reagents in a strongly polar or nonpolar reaction medium with accompanying analytical measurements *via* UV/VIS, IR, and NMR spectroscopy. For example, the reduction with AlMe_3_ performed in printed reaction cuvettes, which led to new compounds and revealed details about the reaction mechanism, demonstrates the usefulness of the method. The concept presented here can easily be extended to further analytical methods and does not require any modifications of the 3D‐printer, the glovebox, or the analytical instruments. The transfer of all necessary equipment inside the glovebox is realized within one night, and reaction flasks and cuvettes are printed within a few hours, allowing the technique to become a complementary routine method for inert‐gas chemistry. Currently, the extension towards further polymers available for 3D‐printing is investigated, to enlarge the spectrum of suitable solvents as well as the use of overpressure.

We thank *G. P. Brunotte* and Dr. *L. Steuernagel* of the Institute of Polymer Materials and Plastics Engineering for the tensile tests, *M. Heinz* and *U. Köcher* of the Institute of Technical Chemistry for SEC and DSC measurements, PD Dr. *J. Adams* of the Institute of Physical Chemistry for fluorescence measurements and *P. Sommer* of the Institute of Mineral and Waste Processing, Waste Disposal and Geomechanics for the ICP‐OES analyses. We thank Dr. *G. Dräger* of the Institute of Organic Chemistry of the Leibniz University Hannover and Dr. *H. Frauendorf* of the Institute of Organic and Biomolecular Chemistry of the Georg‐August University Göttingen for the measurement of the high‐resolution mass spectra.

## Experimental Part

Detailed information of the 3D‐printing setup and the 3D‐printing procedure, as well as additional spectroscopic measurements, further analytics and detailed procedures for experimental setups monitored by UV/VIS, NMR, and IR spectroscopy are given in the *Appendix S1*. STL files of all presented reaction flasks and cuvettes are gladly provided on request.

### General

All operations with air sensitive compounds were carried out at a high‐vacuum line (< 10^−6^ mbar) using *Schlenk* techniques and in a glovebox (*M. Braun*,* Labmaster 130*, M. Braun Inertgas‐Systeme GmbH, Garching, Germany) under N_2_. If not noted differently, all chemicals were bought from *Sigma–Aldrich* (St. Louis, Missouri, USA) and used as received. Cyclohexane and octane (Acros Organics, *Thermo‐Fisher Scientific*, Geel, Belgium) were purchased dry (< 0.002% H_2_O) and used as received. H_2_O was degassed three times before use. All solids used for air‐sensitive operations were degassed for one night at high *in vacuo* and transferred *via* flasks equipped with Teflon stopcocks into the glovebox. AlMe_3_ (*Witco GmbH*, Bergkamen, Germany) was used as received. 1,5‐Diaminopentane was degassed three times before use. Styrene was degassed and dried two times over CaH_2_ and distilled before use. (D_18_)octane (*Deutero GmbH*, Kastellaun, Germany) was degassed and dried with BuLi and distilled before use. Triethylamine was dried two times over CaH_2_ and distilled before use. Cyclohexanone was dried over CaH_2_ and distilled before use. Bromotrimethylsilane was distilled at the vacuum line and only the middle fraction (approx. 80%) was used. Silica gel *60* was used for column chromatography (CC). UV/VIS Spectra: *Jasco V 640* spectrophotometer (*Jasco Germany GmbH*, Gross‐Umstadt, Germany). Fluorescence spectra: *Jasco FP‐8500* fluorescence spectrometer (*Jasco Germany GmbH*). If not noted differently, spectra were recorded with a speed of 100 nm/min. IR Spectra: *Bruker Alpha‐T* FT‐IR spectrometer (*Bruker Coporation*, Billerica, Massachusetts, USA). If not noted differently, 16 spectra were accumulated per measurement. For ATR measurements, a *Platinum diamond‐ATR* unit was used (Bruker Corporation). NMR Spectra: *Bruker Avance 400* (Bruker Corporation) (400 MHz (^1^H), 100 MHz (^13^C) and *Avance III 600* (600 MHz (^1^H), 150 MHz (^13^C) FT‐NMR spectrometer. Chemical shifts are given in ppm relative to tetramethylsilane (*δ *= 0.0) or the residual solvent signal of the deuterated solvent. Mass spectra: *Varian 320 MS TQ* mass spectrometer at 20 eV and 70 eV for EI mass spectra (*Agilent Technologies, Inc*., Santa Clara, CA, USA). Additionally, CI ionization with CH_4_ was applied. High‐resolution mass spectra were measured externally by Dr. *Gerald Dräger* at the Institute of Organic Chemistry of the Leibniz University Hannover and Dr. *Holm Frauendorf* at the Institute of Organic and Biomolecular Chemistry of the Georg August University Göttingen. SEC measurements were performed with a setup equipped with a *Waters 515* HPLC pump (Waters GmbH, Eschborn, Germany), *Knauer Smartline* RI detector 2300 (*Knauer Wissenschaftliche Geräte GmbH*, Berlin, Germany) and 2 × 5 μm mixed‐C and 1 *PLgel 1000* Å column from *Polymer Laboratories* (*Agilent Technologies, Inc*.). THF with a flow rate of 1 ml/min at 25 °C was used as eluent. Molecular weights are given relative to polystyrene calibration. Glass transition temperatures have been measured with a *Mettler‐Toledo DSC‐1* apparatus under N_2_ with a heating speed of 10 K/min (*Mettler‐Toledo GmbH*, Gießen, Germany). ICP‐OES analyses have been performed on a *Varian Vista MPX* instrument in 0.5 vol % HNO_3_ (*Agilent Technologies, Inc*.). Samples have been digested as follows. The pure substance (approx. 5 mg) has been weighed in exactly, dissolved in conc. H_2_SO_4_, and heated 2 h at 140 °C. The solution turned dark brown. After addition of 1.5 ml H_2_O_2_ (30%), heating was continued for 12 h. A quantity of 0.5 ml H_2_O_2_ (30%) was added to the nearly clear solution, and heating was continued for 2 h. The totally colorless solution was cooled down and diluted with 0.5 vol % HNO_3_ to exactly 25.0 ml. Tensile tests were performed with a *Zwick/Roell BZ1‐MM14450.ZW05* (*Zwick GmbH & Co. KG*, Ulm, Germany) universal testing machine equipped with a 10 kN load cell and with a speed of 1 mm/min at 23 °C/50% rel. humidity. A *Heraeus Labofuge 400R* at 20 °C was used for centrifugation (*Thermo Fisher Scientific Messtechnik GmbH*, Oberhausen, Germany). The residual H_2_O content of the polymer samples was measured externally *via Karl–Fischer* titration of the condensed residue emitted from molten polymer samples at 150 °C under N_2_ flow by the ‘*Mikroanalytisches Labor Pascher*’ in Remagen, Germany.

### General Procedure for Reductions with Trimethylaluminium

The reaction vessel combined with an IR cuvette (flask F6) or the reaction flask F2 was printed with natural ABS‐N. The solid starting materials (5 mol‐% *p*‐fluorobenzoic acid as catalyst and approx. 50 – 100 mg of the corresponding alcohol or carboxylic acid) were inserted during a first pause of the print. A soln. of AlMe_3_ (1.000 g, 13.9 mmol) in dry octane (37.870 g) was prepared in a separate flask and used for all reductions. The appropriate amount (5 equiv. AlMe_3_ in case of alcohols, 8 equiv. in case of carboxylic acids) of the TMA stock soln. was added *via* a syringe during the second pause of the 3D‐print, and after cooling down flask F2 or F6 was discharged out of the glovebox. Immediately after discharging, an initial IR spectrum was recorded after turning the flask upside down when flask F6 was used. The reaction flask was heated to 85 °C using an aluminum bead heating bath and IR spectra were recorded every 2 days. After 6 days, the reaction flask was opened with a saw and emptied, the flask washed three times with heptane. H_2_O (50 ml) was added to the combined org. solns. and stirred for 5 min. After phase separation, the aq. phase was extracted three times with heptane (3 × 20 ml), the combined org. phases were dried with Na_2_SO_4_, and the solvent was removed under reduced pressure. For all IR measurements, printed cuvette F5 (ABS‐N) filled with heptane was used as reference cell. Spectra were recorded with a resolution of 2 cm^−1^. Thirty‐two spectra have been accumulated per measurement and the final spectrum has been averaged over 35 data points.

**1,4‐Bis(1,1‐diphenylethyl)benzene** (**3b**). Following the general procedure (flask F2) benzene‐1,4‐diylbis(diphenylmethanol) (62 mg, 0.14 mmol, 0.28 mmol OH) and *p*‐fluorobenzoic acid (2 mg, 5 mol‐%) were treated with TMA solution (6.508 g, 2.3 mmol). The crude product was purified by column chromatography (*R*_f_ (PE/EE 2:1) 0.77) and after removal of the solvent 1,4‐bis(1,1‐diphenylethyl)benzene (**3b**) was obtained. Yield: 27 mg (44%). White solid. M.p. 222 – 224°. IR (ATR): 3083*w*, 3057*w*, 3018*w*, 2975*w*, 2935*w*, 2876*w*, 1596*w*, 1489*w*, 1441*w*, 1023*w*, 1014*w*, 860*w*, 811*w*, 756*m*, 697*s*, 671*w*, 628*m*, 583*m*, 534*w*, 522*w*. ^1^H‐NMR (600 MHz, CD_2_Cl_2_): 2.16 (*s*, 2 Me(1)); 6.99 (*s*, 4 H, H–C(8)); 7.09 – 7.16 (*m*, 8 H, H–C(4)); 7.17 – 7.21 (*m*, 4 H, H–C(6)); 7.22 – 7.31 (*m*, 8 H, H–C(5)). ^13^C‐NMR (150 MHz, CD_2_Cl_2_): 30.8 (C(1)); 52.8 (C(2)); 126.5 (C(6)); 128.4 (C(5)); 128.7 (C(8)); 129.2 (C(4)); 147.2 (C(7)); 149.8 (C(3)). EI‐MS (70 eV): 438 (*M* ^+^, 51), 423 ([*M* – Me]^+^, 100), 408 ([*M* – 2 Me]^+^, 10). HR‐EI‐MS: 438.2350 (C_34_H^+^_30_; calc. 438.2348).

**[Me**_**2**_ **Al(** ***μ*** **‐** ***O*** **‐cedrane)]**_**2**_ (**3d**). Following the general procedure (flask F2) (+)‐cedrol (89 mg, 0.4 mmol) and *p*‐fluorobenzoic acid (3 mg, 5 mol‐%) were treated with TMA solution (5.743 g, 2.1 mmol). The crude product was washed with acetonitrile and after removal of the solvent [Me_2_Al(*μ‐O*‐cedrane)]_2_ (**3d**) was obtained. Yield: 61 mg (28%). White solid. M.p. 152 – 154°. IR (ATR): 3008*w*, 2947*w*, 2931*w*, 2912*w*, 2883*w*, 1456*w* (br.), 1381*w*, 1198*m*, 1112*w*, 1044*w*, 922*m*, 899*s*, 856*w*, 796*w*, 753*m*, 690*s* (Al–C), 671*m*, 643*m*, 566*w*. ^1^H‐NMR (600 MHz, CDCl_3_): −0.63 (br. *s*, 4 Me(13)); 0.83 (*d*,* J *=* *7.1, 2 Me(10)); 0.98 (*s*, 2 Me(12b)); 1.26 (*dddd*,* J *=* *11.8, *J *=* *8.6, 7.7, 6.0, CH_2_(1); 1.31 (*s*, 2 Me(12a)); 1.33 – 1.42 (*m*, CH_2_(2,4,5)); 1.40 (*s*, 2 Me(11)); 1.45 (*dddd*,* J *=* *13.1, 6.4, 2.3, 1.9, CH_2_(5′)); 1.49 – 1.55 (*m*, CH_2_(4′)); 1.60 (*ddd*,* J *=* *12.3, 5.5, 2.9, CH_2_(2′)); 1.64 (*q*,* J *=* *7.0, 2 H, H–C(3)); 1.73 (*dd*,* J *=* *8.1, 2 H, H–C(8a)); 1.74 (*dd*,* J *=* *8.1, 2 H, H–C(7)); 1.88 (*dd*,* J *=* *11.8, 5.5, CH_2_(1′)); 1.88 (*dd*,* J *=* *11.8, 8.1, CH_2_(9)); 2.12 (*ddd*,* J *=* *11.8, 8.1, 8.0, 2 CH_2_(9′)). ^13^C‐NMR (150 MHz, CDCl_3_): −3.6 (C(13)); 15.6 (C(10)); 25.4 (C(5)); 26.9 (C(12b)); 28.5 (C(12a)); 30.7 (C(11)); 31.5 (C(4)); 34.2 (C(9)); 37.1 (C(1)); 41.2 (C(3)); 41.4 (C(2)); 43.1 (C(8)); 52.9 (C(3a)); 57.9 (C(8a)); 60.7 (C(7)); 81.8 (C(6)). ^1^H‐NMR (600 MHz, (D_8_)toluene, 70°): −0.32 (*s*, 4 Me(13)); ^13^C‐NMR (150 MHz, (D_8_)toluene, 70°): −2.9 (C(13)). CI‐MS: 441 ([*M* – Me]^+^, 90), 337 (*M* – Me – cedrane]^+^, 15), 205 (cedrane^+^, 100). HR‐EI‐MS: 541.4147 (C_33_H_59_Al_2_O^+^_2_, [*M* – Me]^+^; calc. 541.4151). ICP‐OES: Al 10.19% (C_34_H_62_Al_2_O_2_; calc. 9.69%).

Details for the synthesis of 1,1,1‐triphenylethane (**3b**), [Me_2_Al(*μ‐O*‐adamantyl)]_2_ (**3d**) and the reduction of 4‐butylbenzoic acid are given in the *Appendix S1*.

### Reaction of Cyclohexanone with Trimethylsilyl Bromide

The reaction flask F8 combined with a printed NMR tube was printed with natural ABS‐N. A stock solution of dry cyclohexanone (122 mg, 1.2 mmol) in dry (D_18_)octane (1.602 g) was prepared separately. A solution of freshly distilled trimethylsilyl bromide (TMSBr) (258 mg, 1.7 mmol, 3.2 eq.) in (D_18_)octane (738 mg) was mixed with the cyclohexanone stock solution (733 mg, 0.5 mmol). The combined clear solution was inserted to flask F8 *via* a syringe during a first pause of the print. Dry triethylamine (184 mg, 1.8 mmol, 3.4 eq.) was added *via* a microsyringe during a second pause of the print. After finishing the print, flask F8 was discharged from the glovebox and carefully checked for irregularities. Initial NMR spectra (^13^C, ^29^Si) were recorded directly after discharging and flask F8 was heated upside down at 85 °C in an aluminum bead bath. The flask was cooled down for the repeated measurement of NMR spectra and heating continued after each measurement. After completion of the reaction, flask F8 was opened and the reaction solution transferred into a NMR tube for a final measurement. All NMR spectra have been recorded on the *Bruker Avance III 600* spectrometer without spinning the spinner/tube combination (flask F8). For all acquisitions a deuterium lock on the (D_18_)octane signal has been applied. Before each measurement, the automatic shimming routine was performed. A flip angle of 30° (^13^C: zgpg30, ^29^Si: zgig30) and a relaxation delay of D1 = 2 s (^13^C), D1 = 0.2 s (^29^Si) has been used. A total of 512 measurements have been accumulated for each FID. Exponential multiplication with a line broadening (lb) of factor 2 has been applied. Spectra are referenced to the C(2)‐carbon of (D_18_)octane at 22.0 ppm (^13^C) or TMSBr at 24.3 ppm (^29^Si) which was measured in the presence of tetramethylsilane in (D_18_)octane separately.

### General Procedure for Polymerization

The reaction flask F2 was printed with natural ABS‐N. The solid educts (CuBr (approx. 0.175 mmol), 4,4′‐dinonyl‐2,2′‐bibyridine (dnnbipy) (approx. 0.350 mmol) were inserted during a first pause of the print. A solution of styrene (5.368 g, 51.5 mmol) in dry cyclohexane (42.436 g, 0.504 mol) was prepared in a separated flask and used for all polymerizations. The appropriate amount (approx. 16.2 g stock solution, 17.5 mmol styrene, 0.17 mol cyclohexane) of the stock solution was transferred to a mixing flask; dodecyl 2‐bromoisobutyrate (dbib) (approx. 0.035 mmol) was added *via* a microsyringe and the solution shaken intensively. The mixture of initiator and styrene was added to flask F2 *via* a syringe during the second pause of the 3D‐print and after finishing the print flask F2 was discharged out of the glovebox. The reaction flask was heated to 85 °C using an aluminum bead heating bath. After heating up, the flask was held well above the upper critical solution temperature of polystyrene in cyclohexane (35 °C) until precipitation of the polymer. After the desired reaction time, the warm reaction flask was opened with a saw, emptied, the flask washed two times with warm cyclohexane and the combined solutions precipitated in 160 ml MeOH. After centrifugation for 15 min (2800*g*), the supernate solution was decanted, the residue dissolved in 5 ml (*R*)‐limonene *via* sonication, filtered through a 0.45 μm syringe filter and precipitated in 160 ml MeOH. After centrifugation, the white residue was dried *in vacuo*.

## Supporting information


**Appendix S1.** 3D‐Printing inside the glovebox: A versatile tool for inert gas chemistry combined with spectroscopy.
