# Supplementary material for: 3D‐Printing inside the Glovebox: A Versatile Tool for Inert‐Gas Chemistry Combined with Spectroscopy
Source: Helv Chim Acta. 2016 Apr 22;99(4):255–66. doi: 10.1002/hlca.201500502 (PMC4840480; doi:10.1002/hlca.201500502)
Supplement: Supplementary file 1 — Appendix S1. 3D‐Printing inside the glovebox: A versatile tool for inert gas chemistry combined with spectroscopy. [file HLCA-99-255-s001.pdf]

Supporting Information:

3D-printing inside the glove-box: A versatile tool for inert gas chemistry combined with spectroscopy

by **Felix Lederle**[a]), **Christian Kaldun**[a]), **Jan C. Namyslo**[a]) and **Eike G. Hübner***[a])

[a]) Clausthal University of Technology

Institute of Organic Chemistry

Leibnizstr. 6

38678 Clausthal-Zellerfeld

Germany

E-mail: eike.huebner@tu-clausthal.de

Table of Contents:

1. 3D-printing 3

2. Detailed experimental procedures 8

3. NMR-spectra of reaction products 16

4. References 22

1. 3D-printing

The UP Plus 2 3D-printer from TierTime Technology Co. Ltd. (PP3DP) was used for all prints without modifications. The printer fits into the standard vacuum chamber of common glove boxes (39 cm diameter) and was evacuated together with all necessary equipment for 1 night before insertion into the glove box. The printing platforms were pasted with ScotchBlue tape (3M) before every print outside the glove box and several platforms kept on stock inside the box. To enable the USB communication with the printer without additional cable connections leading into the glove box, the USB signal was transmitted *via* ethernet and the ethernet connection was realized by PowerLAN. Outside of the Box the ethernet signal was converted by a standard PowerLAN adapter connected to the power supply of the glove box for the balance and other electronic devices inside the glove box. Inside the glove box the ethernet signal was converted back by a second PowerLAN adapter and passed to an USB over Ethernet Server (UE204, B&B Electronics) which allowed the connection of the 3D-printer. UP! Software 2.13 was used for all prints. The platform was levelled before each print and preheated for 15 min. Printing was performed with a layer height of 0.15 mm and "fine" printing settings. Deviating from the standard parameters, a hatch width of 0.32, hatch speed of 30, jump speed of 50 and hatch scale of 1.0 was set to obtain solid walls.[1] These parameters are only valid for a wall thickness of ~ 3 mm and changes may be necessary for other structures and wall thicknesses. Flasks F3, F5 and F7 printed at air were printed with standard printing settings. Nozzle temperature and all other parameters remained unchanged. Printing was paused shortly and the platform moved to the front to insert solvents and reagents and the stopping time kept as short as possible (few seconds). Printing was continued and if necessary further stops for the addition of subsequent reagents conducted. After completion of the print, a slow cooling down procedure was performed by cooling down for 10 K and heating up for 5 K until a platform temperature of 50 °C was reached. Reaction vessels were discharged from the box and checked for irregularities. Caution has to be taken in all cases with pyrophoric substances and flammable solvents before heating the reaction vessels. Extreme careful control of printed NMR spinner/tube-combinations should be performed to ensure correct fitting inside the NMR. Heating was performed in a heating bath of aluminium beads (Lab Armor) to prevent contamination of printed cuvettes. Reaction vessels were opened with a saw after completion of the reaction. Pressure equalization was hearably noticeable during opening of the reaction vessels for some reactions with trimethylaluminium.
3D-models of all reaction vessels and cuvettes were constructed with SketchUP Make 15.3.330. Models were checked and repaired with Netfabb basic 5.2.1.

The 3D-printing materials ABS (natural, ABS-N), ABS (clear, ABS-K) and smartABS (natural) were bought as 1.75 mm filaments from Orbi-Tech GmbH, Leichlingen and dried over night at 60 °C and left one night in the vacuum chamber before insertion into the glove box.

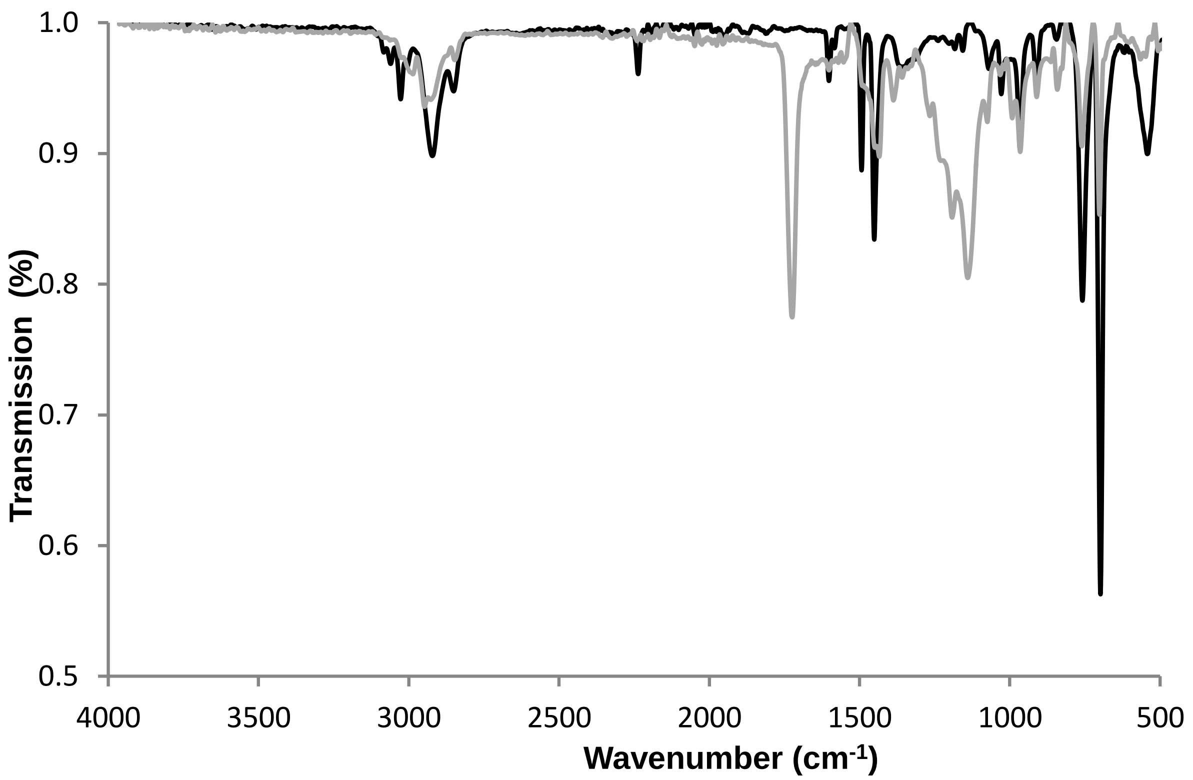

Figure S1: ATR IR spectra of samples of ABS-N (black) and ABS-K (grey) after evacuation for 1 night in the vacuum chamber.

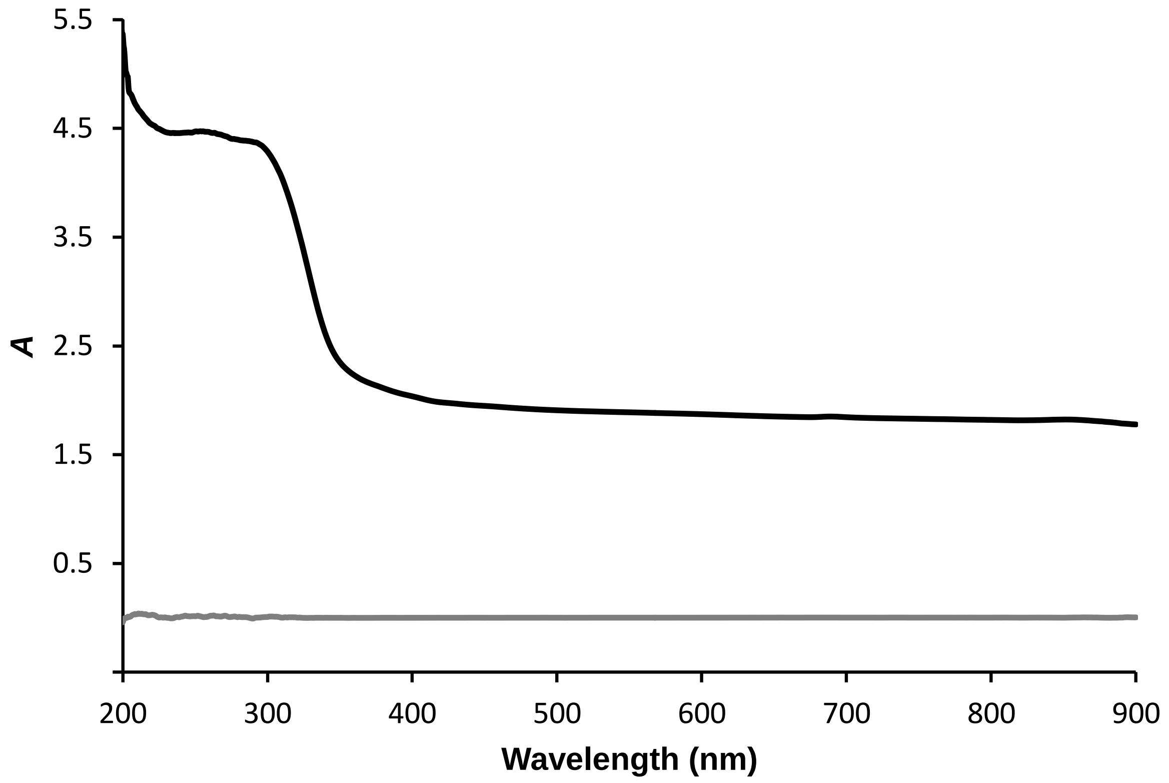

Figure S2: UV/VIS spectra of cuvette F3 (ABS-K) vs. air (black) and vs. cuvette F3 (ABS-K) (grey).

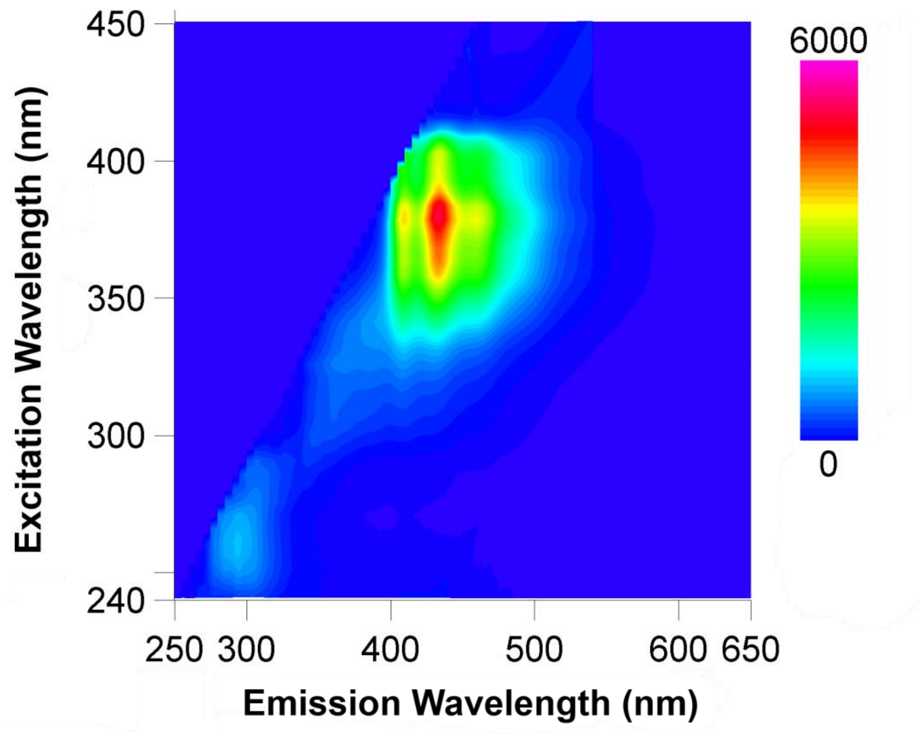

Figure S3: Excitation-emission matrix of a plate of ABS-K measured at an angle of 45°.

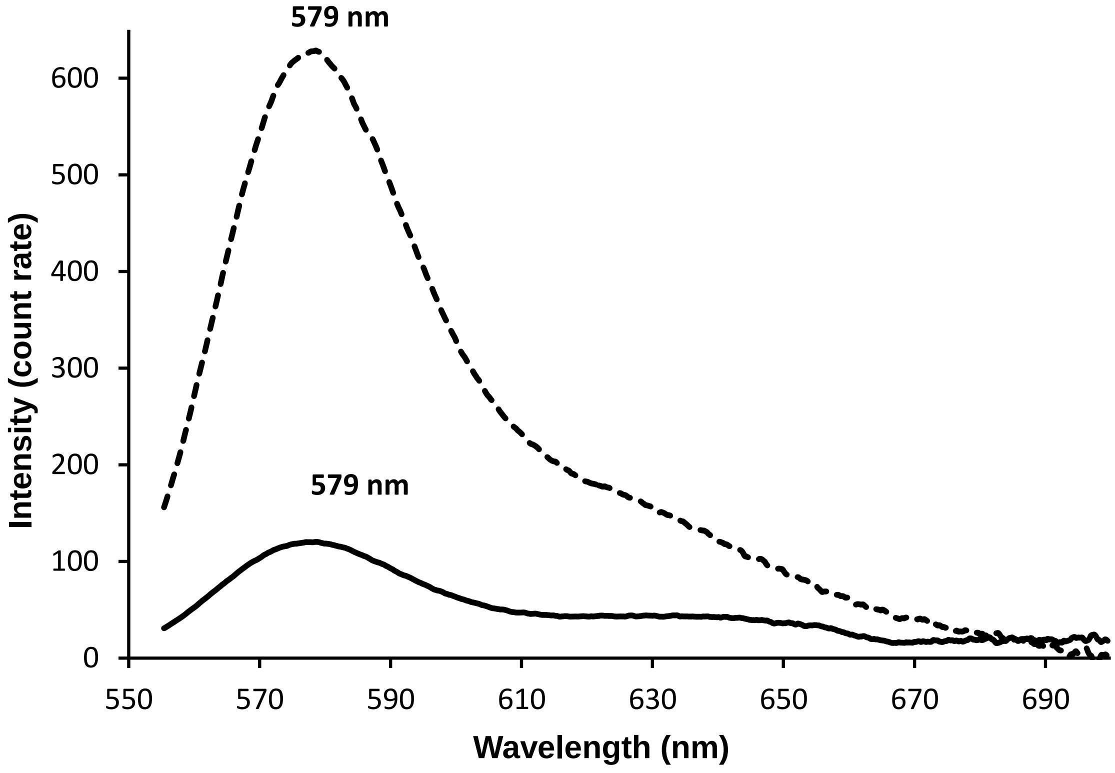

Figure S4: Emission spectra of rhodamine B (0.001 mM) in water in a glass cuvette (dashed line) and cuvette F3 (solid line) at an excitation wavelength of λ_{exc}=545 nm.

The soluble fraction of the polymers has been isolated as follows. ABS-N (3.00 g) and ABS-K (3.00 g) have been weighed in separately and dissolved in acetone overnight. After centrifugation for 24 h (4500 U min^{-1}) the supernatant solution has been filtered through a 0.45 µm syringe filter. The solvent has been removed in vacuo and the clear residue dried for 5 days at high vacuum. The soluble residue was determined to:

ABS-N: 2.342 g, 78 %.

ABS-K: 1.899 g, 63 %.

The soluble fractions have been analyzed *via* SEC.

ABS-N: M_n = 67 000 g mol^{-1}; PDI = 2.25

ABS-K: M_n = 55 000 g mol^{-1}; PDI = 2.08

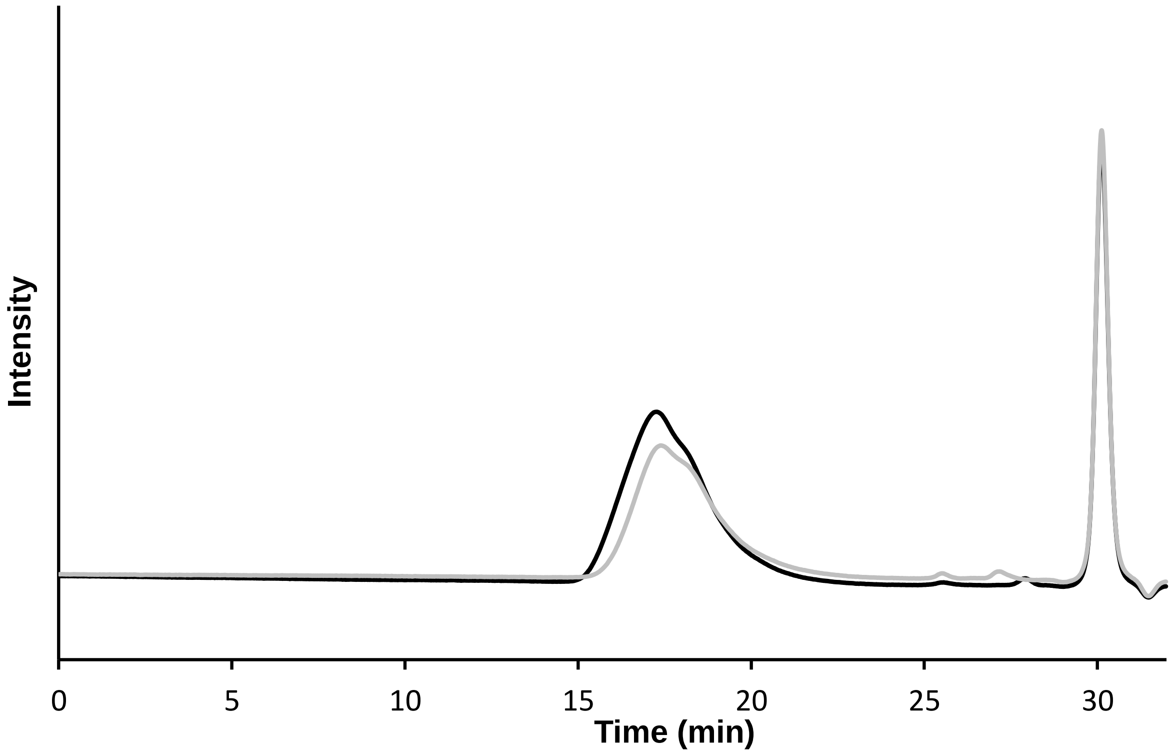

Figure S5: SEC traces of samples of the soluble part of ABS-N (black) and ABS-K (grey).

Test specimens for tensile testing have been dimensioned according to EN ISO 527-2 (type 1BB, 30×4×2 mm) as well as small stripes (30×10×3 mm). After printing each set of samples (longest side orthogonal to the printing platform and shortest side parallel to the moving direction of the printing platform) out of ABS-N (3 samples per type) inside the glove box and outside (at normal air) with the printing settings for solid walls as mentioned above (and a pause of 6 s between the layers for the specimen 1BB) tensile tests have been performed.

The parameters were evaluated to:

Sample	E (Elastic modulus)	σ_M (tensile strength)	ε_B (elongation at break)
1BB – air	1.56 ± 0.07 kN mm^{-2}	25.5 ± 0.9 N mm^{-2}	2.4 ± 0.5 %
1BB – glove box	1.47 ± 0.06 kN mm^{-2}	26.9 ± 1.6 N mm^{-2}	3.1 ± 0.4 %
Plate – air	0.87 ± 0.03 kN mm^{-2}	19.4 ± 0.8 N mm^{-2}	4.4 ± 1.5 %
Plate – glove box	0.85 ± 0.04 kN mm^{-2}	21.6 ± 0.2 N mm^{-2}	10.7 ± 2.6 %

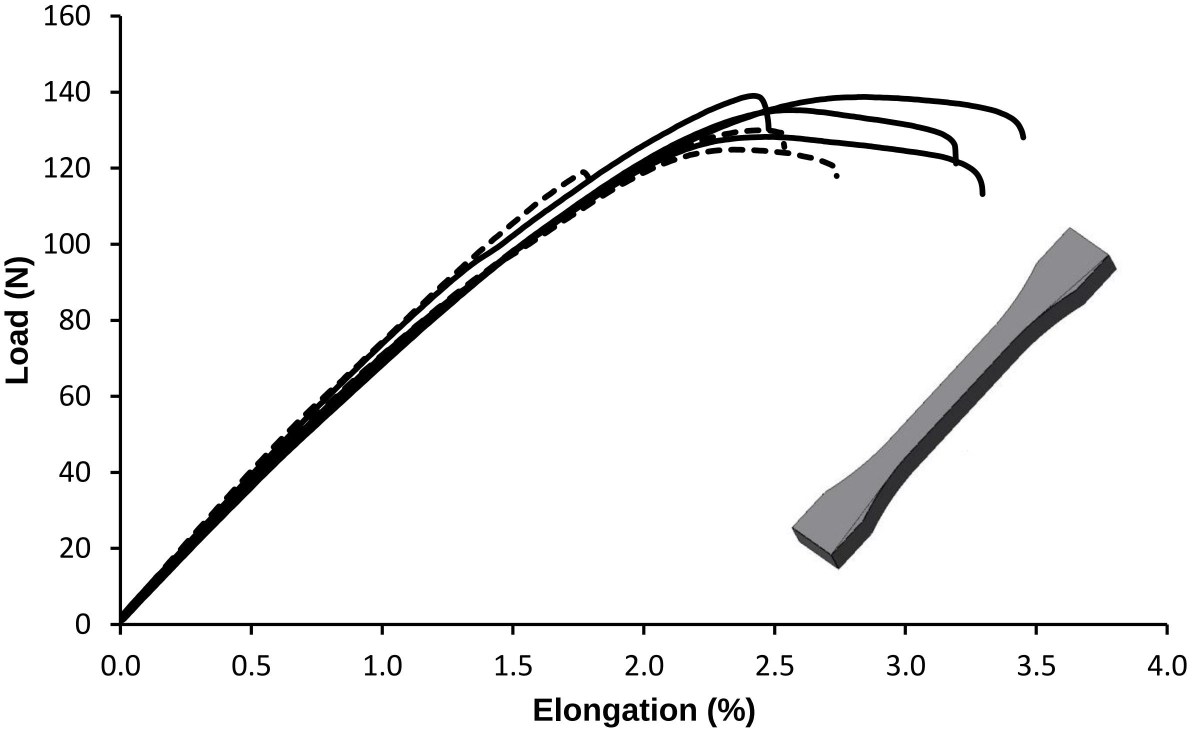

Figure S6: Load-strain curves for test specimen 1BB (30×4×2 mm) printed at air (dotted line) and under nitrogen atmosphere (black line).

2. Detailed experimental procedures

2.1 Reaction of pyrogallol with oxygen (control of the permeability against oxygen)

The reaction vessel combined with an UV/VIS cuvette (F4) was printed with clear ABS-K. A mixture of pyrogallol (0.635 g, 5.0 mmol) and NaOH (0.822 g, 20.6 mmol) was dissolved in degassed water (30.0 mL) and stirred for 15 min. The solution turned slightly yellow during solvation. 10.0 mL of the solution were transferred into flask F4 *via* a syringe during a single pause of the 3D-print and after cooling down flask F4 was discharged out of the glove box. UV/VIS spectra were recorded daily after shaking of the reaction vessel and turning flask F4 over head. After 6 days, flask F4 was punctured by a hot needle near the bottom of the turned flask and UV/VIS spectra were recorded every 2 min for 16 min and after 1 h. The bottle was shaken slightly between the measurements.

For all UV/VIS measurements printed cuvette F3 (ABS-K) filled with deionized water was used as reference cell. Spectra were recorded with a speed of 1000 nm min^{-1}.
Due to changes in the absorption level in dependence of the exact positioning of flask F4 all spectra were linearly shifted to an absorption of 0 at 700 nm.

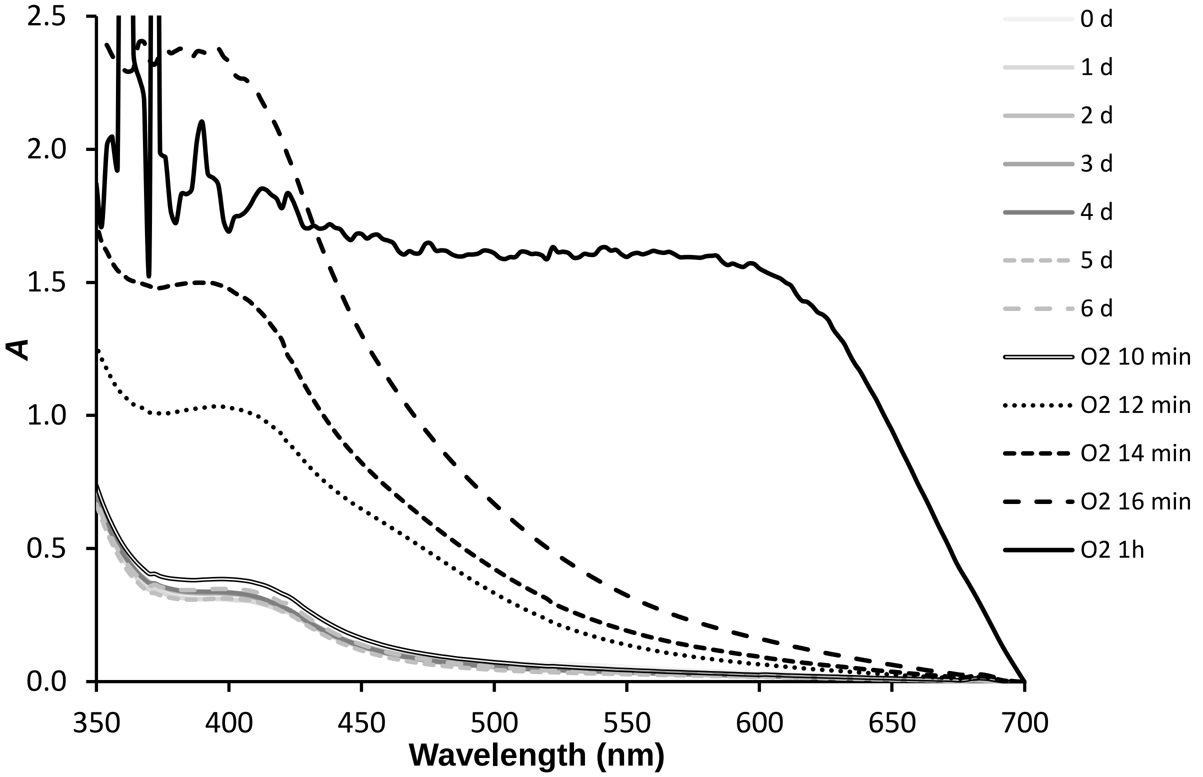

Figure S7: UV/VIS spectra of the reaction solution measured daily and after opening.

2.2 Reaction of CuCl with oxygen

The reaction vessel combined with an UV/VIS cuvette (flask F4) was printed with clear ABS-K. CuCl (53 mg, 0.5 mmol) was inserted during a first pause of the print. 1,5-Diaminopentane (0.221 g, 2.2 mmol) was dissolved in degassed water (20.0 mL) and stirred for 15 min. 10.0 mL of this solution were transferred into flask F4 *via* a syringe during a second pause of the 3D-print and after cooling down flask F4 was discharged out of the glove box. UV/VIS spectra were recorded daily after intense shaking of the reaction vessel and turning flask F4 over head. After the first day, a formation of solid copper at the bottom wall of the flask was clearly noticeable. After 6 days, flask F4 was punctured by a hot needle near the bottom of the turned flask and UV/VIS spectra were recorded every 2 min for 22 min. The bottle was shaken slightly between the measurements.

For all UV/VIS measurements printed cuvette F3 (ABS-K) filled with deionized water was used as reference cell. Spectra were recorded with a speed of 1000 nm min^{-1}.
Due to changes in the absorption level in dependence of the exact positioning of flask F4 all spectra were linearly shifted to the same absorption at 440 nm.

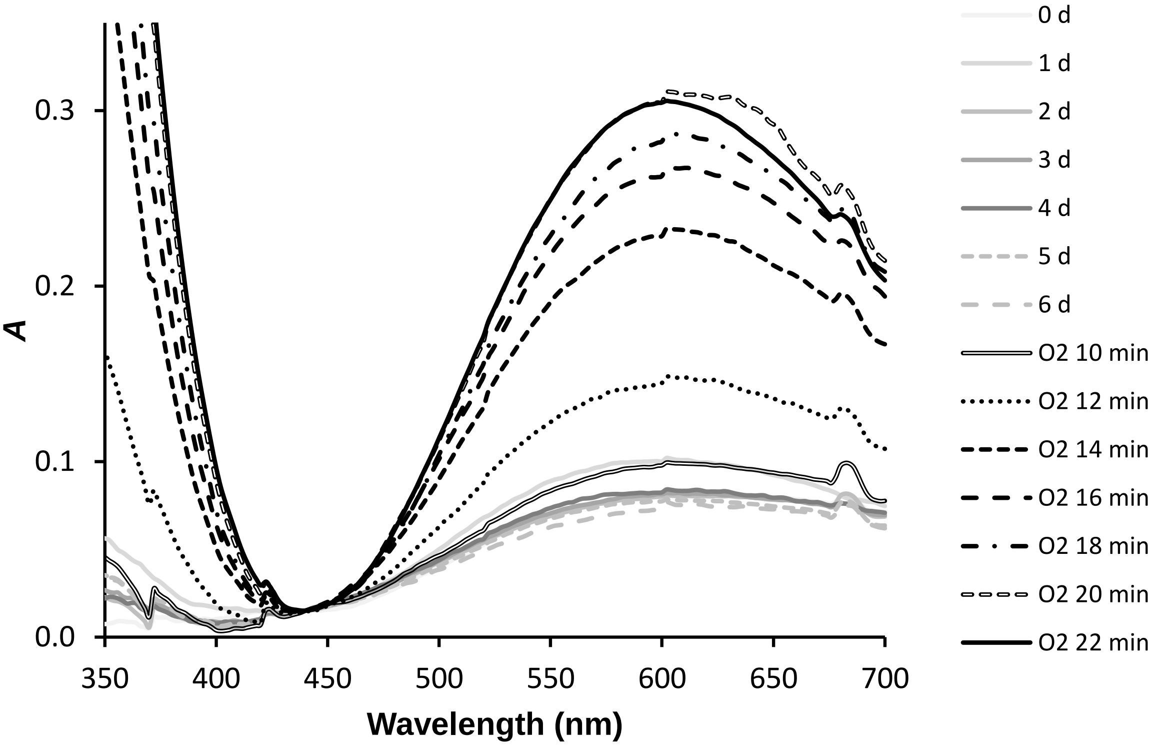

Figure S8: UV/VIS spectra of the reaction solution measured daily and after opening.

2.3 Reactions with trimethylaluminium

2.3.1 Reduction of triphenylmethanol (**2a**)

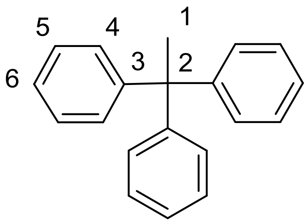

Following the general procedure (flask F6) triphenylmethanol (102 mg, 0.4 mmol) and *p*-fluorobenzoic acid (3 mg, 6 mol-%) were treated with TMA solution (5.645 g, 2.0 mmol). The initial IR spectrum showed the absence of any OH signals. The crude product was purified by column chromatography (PE:EE, 2:1 v/v, R_f=0.84) and after removal of the solvent 1,1,1-triphenylethane (**3a**) was obtained as white solid.[2,3] Yield: 69 mg (69 %).

^{1}H NMR (400 MHz, $CDCl_3$ = 7.26 ppm) δ=2.19 (s, 3H, H-1), 7.06-7.31 (m, 15H, H-Ar); **^{13}C NMR** (100 MHz, $CDCl_3$ = 77.0 ppm) δ=30.5 (C-1), 52.5 (C-2), 125.9 (C-6), 127.8 (C-5), 128.7 (C-4), 149.0 (C-3).

2.3.2 Reduction of (*p*)-*n*-butylbenzoic acid (**2e**)

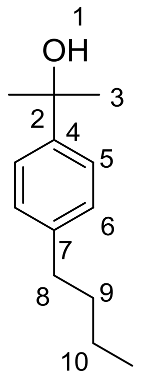

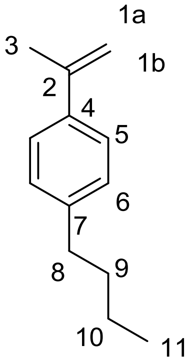

Following the general procedure (flask F6) (*p*)-*n*-butylbenzoic acid (75 mg, 0.4 mmol) was treated with TMA solution (9.980 g, 3.6 mmol). The crude product was analyzed *via* NMR spectroscopy and identified as a mixture of 2-(*p*-(*n*-butylphenyl))propan-2-ol (**3e**) and (*p*)-*n*-butyl-α-methylstyrene (**4e**) in the ratio 2.6:1 and not further purified.[4,5] Yield: 51 mg (47 % 2-(*p*-(*n*-butylphenyl))propan-2-ol (NMR)).

3e: ^{1}H NMR (400 MHz, $CDCl_3$ = 7.26 ppm) δ=0.93 (t, $^3J_{H,H}$=7.4 Hz, 3 H, H-11), 1.31 – 1.42 (m, 2 H, H-10), 1.54 – 1.63 (m, 2 H, H-9), 1.58 (s, 6 H, H-3), 2.60 (t, $^3J_{H,H}$=7.8 Hz, 2 H, H-8), 7.16 (d, $^3J_{H,H}$=8.3 Hz, 2 H, H-6), 7.40 (d, $^3J_{H,H}$=8.3 Hz, 2 H, H-5); **^{13}C NMR** (100 MHz, $CDCl_3$ = 77.0 ppm) δ=14.0 (C-11), 22.4 (C-10), 31.7 (C-3), 33.6 (C-9), 35.2 (C-8), 72.4 (C-2), 124.3 (C-5), 128.2 (C-6), 141.4 (C-7), 146.4 (C-4).

4e: ^{1}H NMR (400 MHz, $CDCl_3$ = 7.26 ppm) δ=0.88 (t, $^3J_{H,H}$=7.0 Hz, 3 H, H-11), 2.14 (m, 3 H, H-3), 5.03 (m, 1 H, H-1a), 5.34 (m, 1 H, H-1b), 7.14 (d, $^3J_{H,H}$=8.3 Hz, 2 H, H-6), 7.39 (d, $^3J_{H,H}$=8.3 Hz, 2 H, H-5) **^{13}C NMR** (100 MHz, $CDCl_3$ = 77.0 ppm) δ=14.1 (C-11), 21.9 (C-3), 22.4 (C-10), 33.6 (C-9), 35.3 (C8), 111.6 (C-1), 125.4 (C-5), 128.3 (C-6).

The signals of H-10, H-9 and H-8 are masked by the signals of **3e**. The quaternary carbon atoms C-2, C-4 and C-7 could not be identified in the mixture.

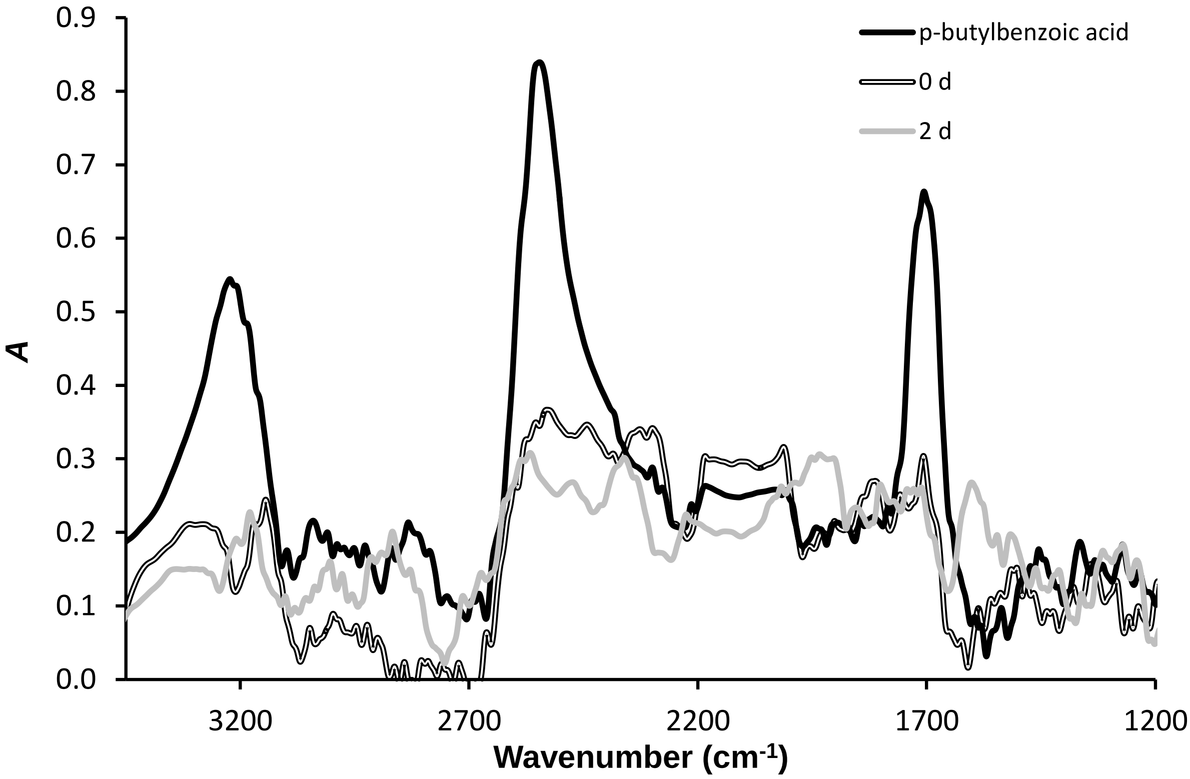

Figure S10: IR spectra of the reaction educt (*p*)-*n*-butylbenzoic acid in *n*-heptane (black) and the reaction mixture directly after discharging flask F6 from the glove box (double line) and after 2 days at 85 °C (grey).

2.3.3 Reaction of 1-adamantol (**2c**)

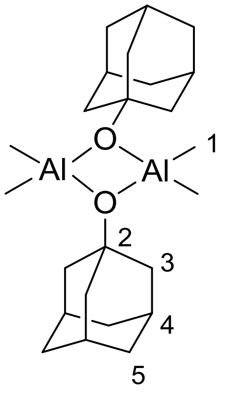

Following the general procedure (flask F2) 1-adamantol (63 mg, 0.4 mmol) and *p*-fluorobenzoic acid (3 mg, 5 mol-%) were treated with TMA solution (6.351 g, 2.3 mmol). The crude product was recrystallized from dry chloroform to yield [Me_2Al(μ-O-adamantyl)]$_2$ (**3c**) as a white powder.[6] Yield: 57 mg (33 %).

^{1}H NMR (600 MHz, $CDCl_3$ = 7.26 ppm) δ=–0.73 (s, 12H, H-1), 1.60 (b, 12 H, H-5), 1.85 (b, 12 H, H-3), 2.14 (b, 6 H, H-4) ppm; **^{13}C NMR** (150 MHz, $CDCl_3$ = 77.0 ppm) δ=–6.0 (C-1), 30.8 (C-4), 35.8 (C-5), 45.4 (C-3), 73.7 (C-2) ppm; **IR** (ATR): $\tilde{\nu}$= 2914 (m,b), 2850 (m), 1451 (w), 1345 (w), 1302 (w), 1192 (w), 1116 (w), 1102 (w), 1087 (w), 1063 (w), 977 (w), 926 (w), 813 (w), 780 (w), 734 (m), 681 (vs, Al-C), 633 (m), 531 (m) cm^{-1}; **MS** (EI, 20 eV): *m/z* (%): 401 (100) [M$^+$–Me], 251 (5) [M$^+$–Me –O-adamantyl], 135 (25) (adamantane$^+$ –H).

2.4 NMR experiments

2.4.1 Reaction of cyclohexanone with trimethylsilyl chloride

The reaction flask F8 combined with an NMR-tube was printed with natural ABS-N. Stock solutions of dry cyclohexanone (101 mg, 1.0 mmol) in dry d18-octane (1.311 g) and of distilled trimethylsilyl chloride (TMSCl) (198 mg, 1.8 mmol) in d18-octane (1.362 g) were prepared separately. The TMSCl stock solution (845 mg, 1.0 mmol, 1.6 eq.) was mixed with the cyclohexanone stock solution (857 mg, 0.6 mmol). The combined clear solution was inserted to flask F8 *via* a syringe during a first pause of the print. Dry triethylamine (131 mg, 1.3 mmol, 2.0 eq.) was added *via* a microsyringe during a second pause of the print. After finishing the print, flask F8 was discharged from the glove box and carefully checked for irregularities. Initial NMR-spectra (^{13}C, ^{29}Si) were recorded directly after discharging and flask F8 was heated upside down at 85 °C in a aluminum bead bath. The flask was cooled down for the repeated measurement of NMR-spectra and heating continued after each measurement.

No indication of enolisation was noticeable in the NMR-spectra.

2.4.2 Reaction of cyclohexanone with trimethylsilyl bromide

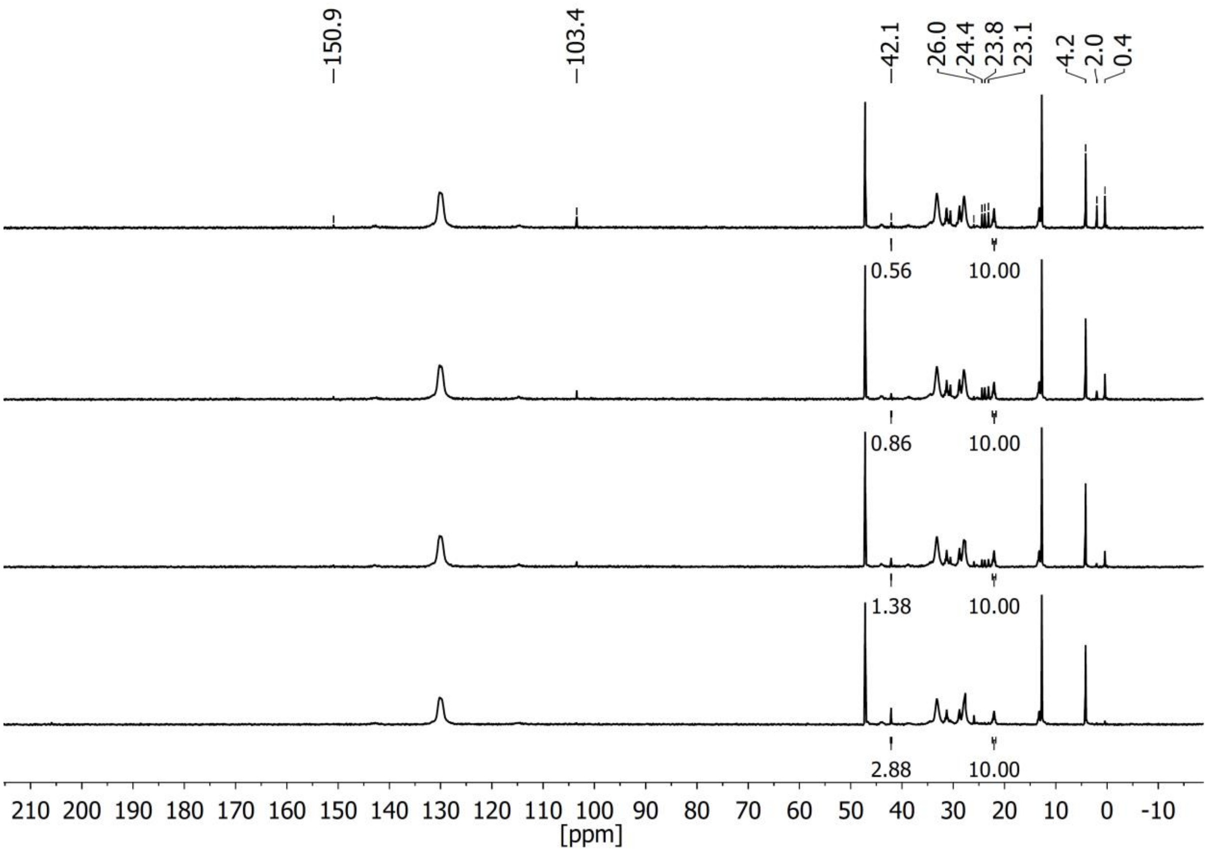

Figure S11: ^{13}C-NMR-spectra of the enolisation of cyclohexanone with TMSBr directly after discharging flask F8 from the glove box (bottom) and after 120, 210 and 300 min (top) at 85 °C.

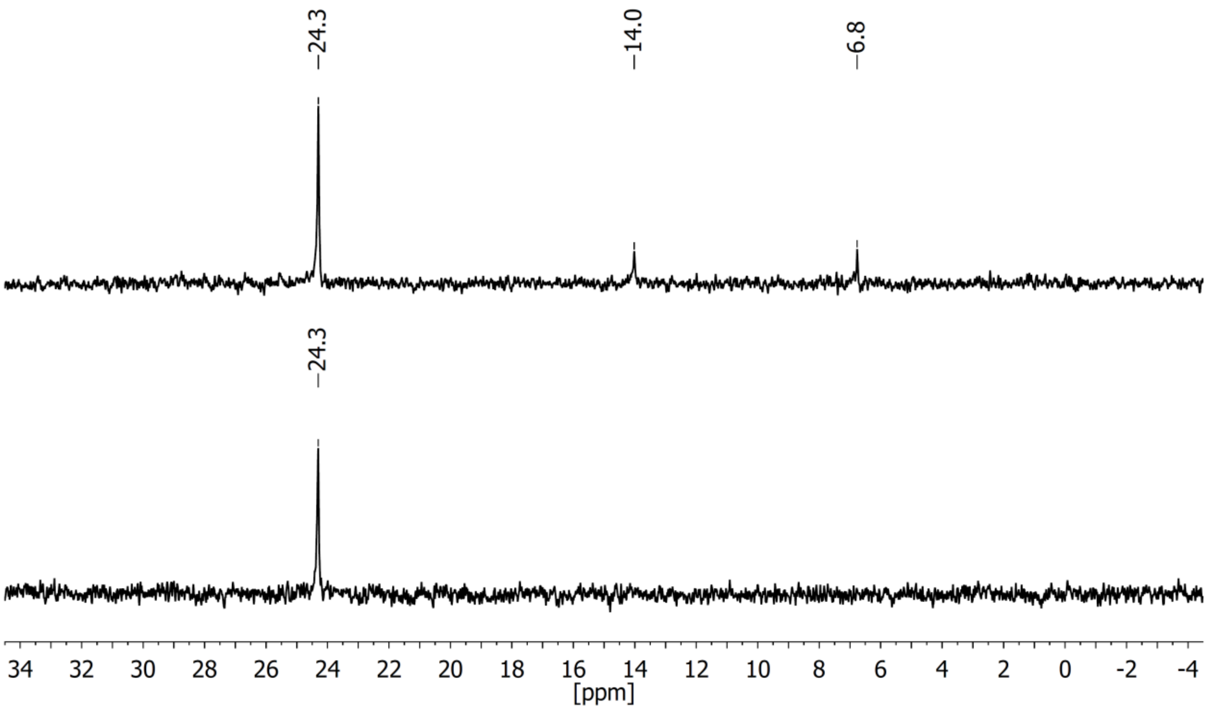

Figure S12: ^{29}Si-NMR-spectra of the enolisation of cyclohexanone with TMSBr directly after discharging flask F8 from the glove box (bottom) and after 300 min (top) at 85 °C.

2.5 Polymerisation

2.5.1 Polystyrene **P1**

Following the general procedure, CuBr (23 mg, 0.16 mmol) and dnnbipy (143 mg, 0.35 mmol) were used as catalyst. Dbib (10 mg, 0.03 mmol) as initiator was mixed with the styrene stock solution (15.799 g, 17.0 mmol styrene) and added to the catalyst. After 21 h at 85 °C the polymer was precipitated as white powder.

Yield: 51 mg.
The molecular weight was determined *via* SEC: M_n = 3600 g mol^{-1}; PDI = 1.06.

2.5.2 Polystyrene **P2**

Following the general procedure, CuBr (27 mg, 0.19 mmol) and dnnbipy (142 mg, 0.35 mmol) were used as catalyst. Dbib (10 mg, 0.03 mmol) as initiator was mixed with the styrene stock solution (15.323 g, 16.5 mmol styrene) and added to the catalyst. After 56 h at 85 °C the polymer was precipitated as white powder.

Yield: 77 mg.
The molecular weight was determined *via* SEC: M_n = 4700 g mol^{-1}; PDI = 1.08.

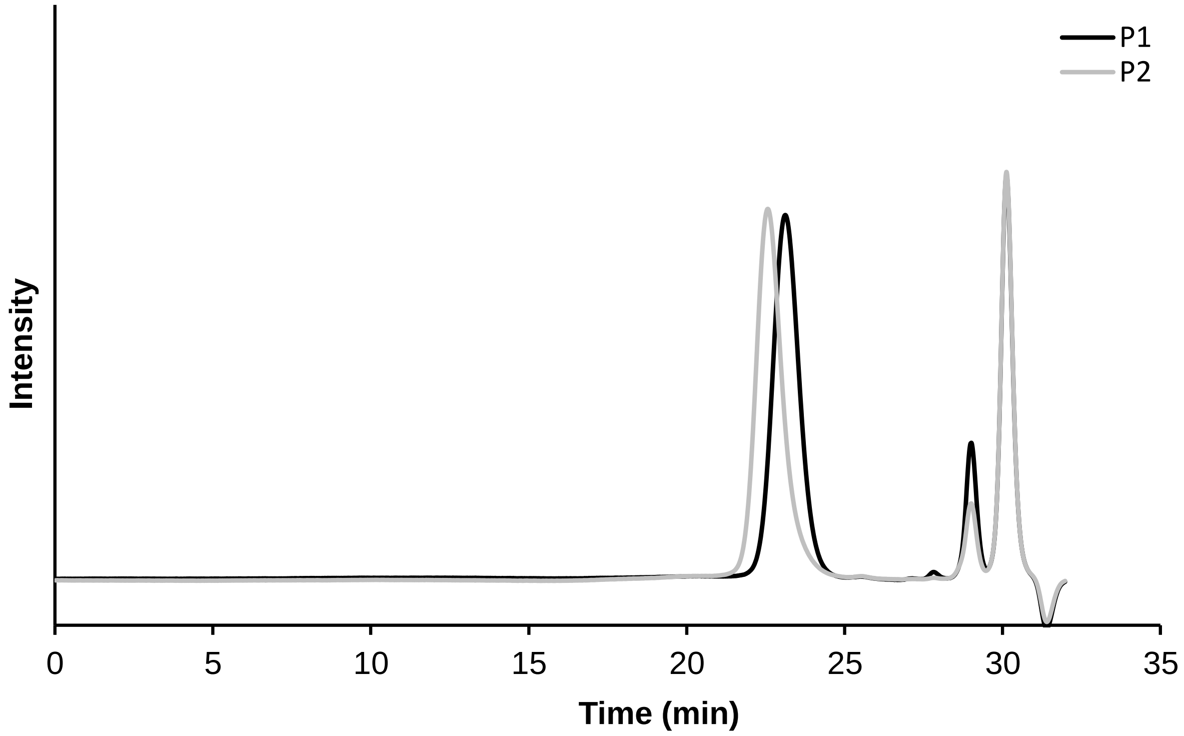

Figure S13: SEC traces of the polymer samples **P1** (black) and **P2** (grey).

3. NMR-spectra of reaction products (^1H (top) and ^{13}C (bottom))

NMR-spectra for 1,1,1-triphenylethane.

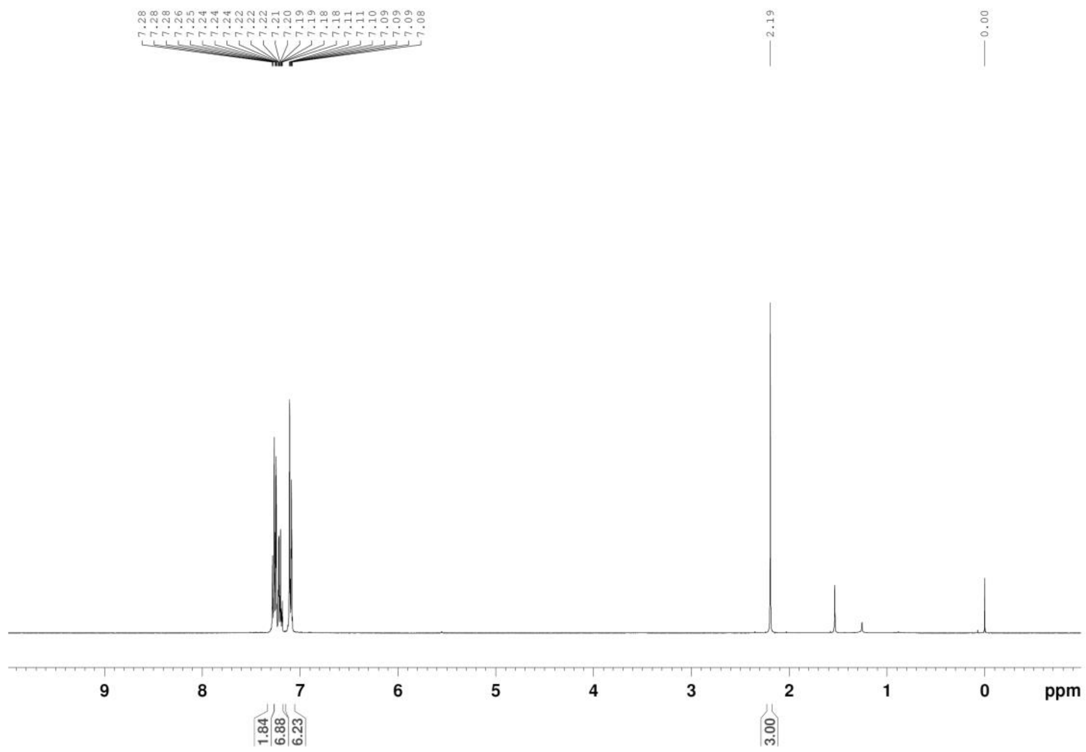

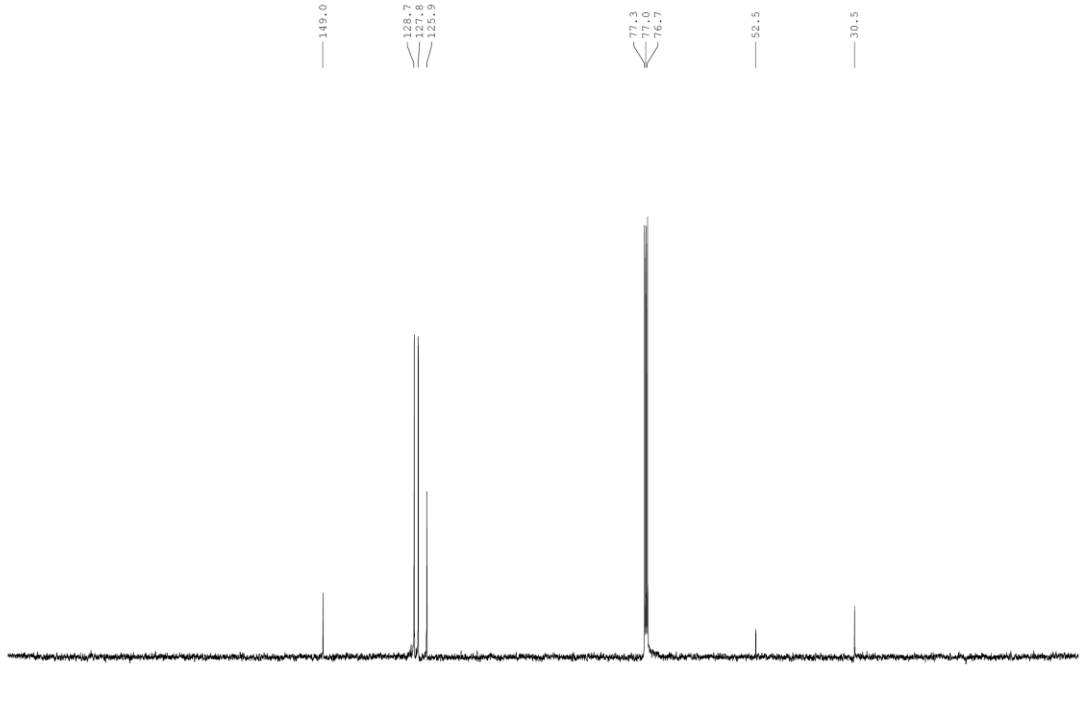

NMR-spectra for 1,4-bis(1,1-diphenylethyl)benzene.

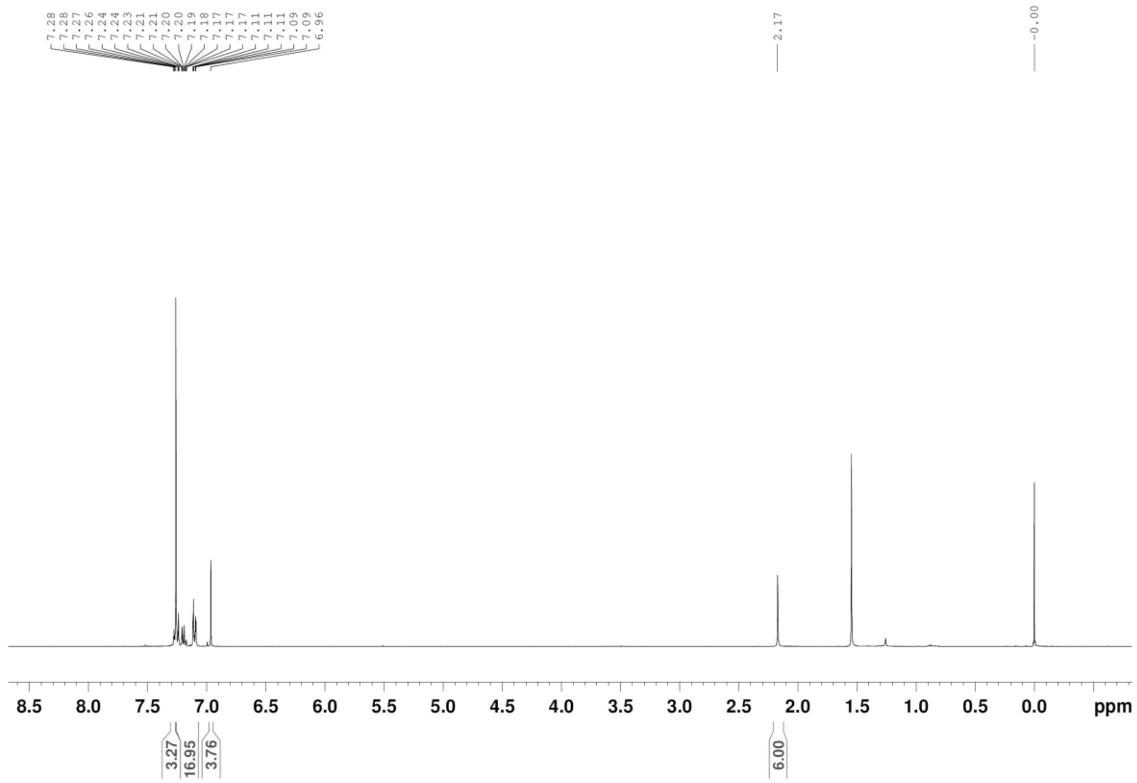

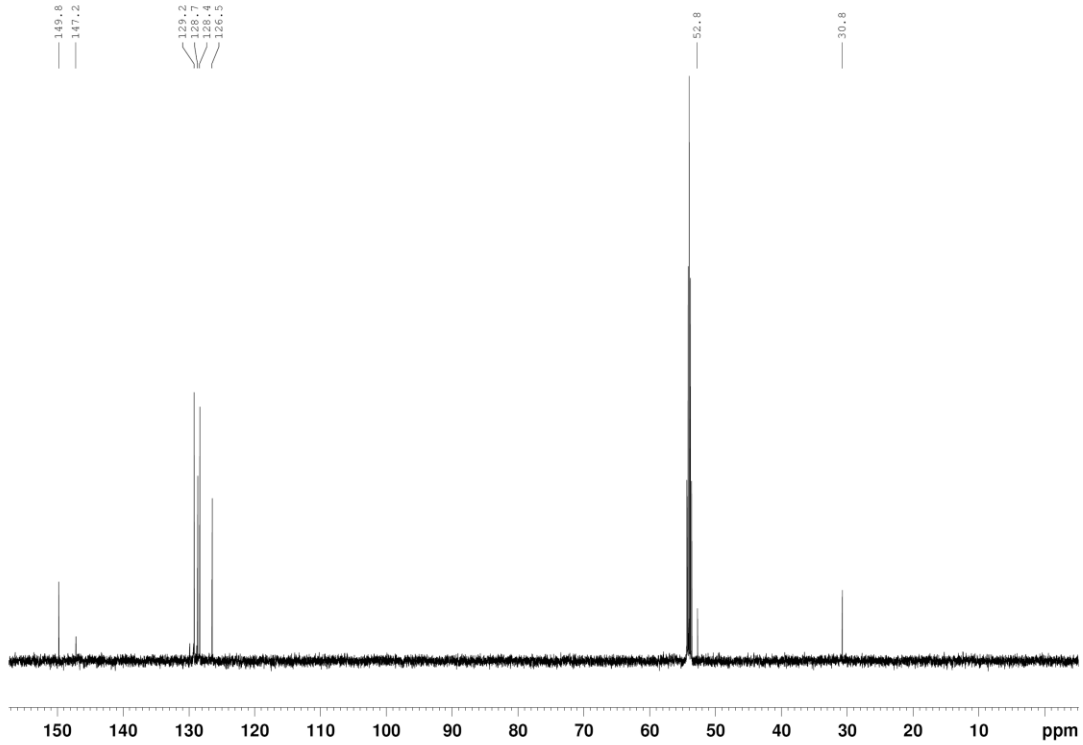

NMR-spectra for 2-(*p*-(*n*-butylphenyl))propan-2-ol and (*p*)-*n*-butyl-α-methylstyrene.

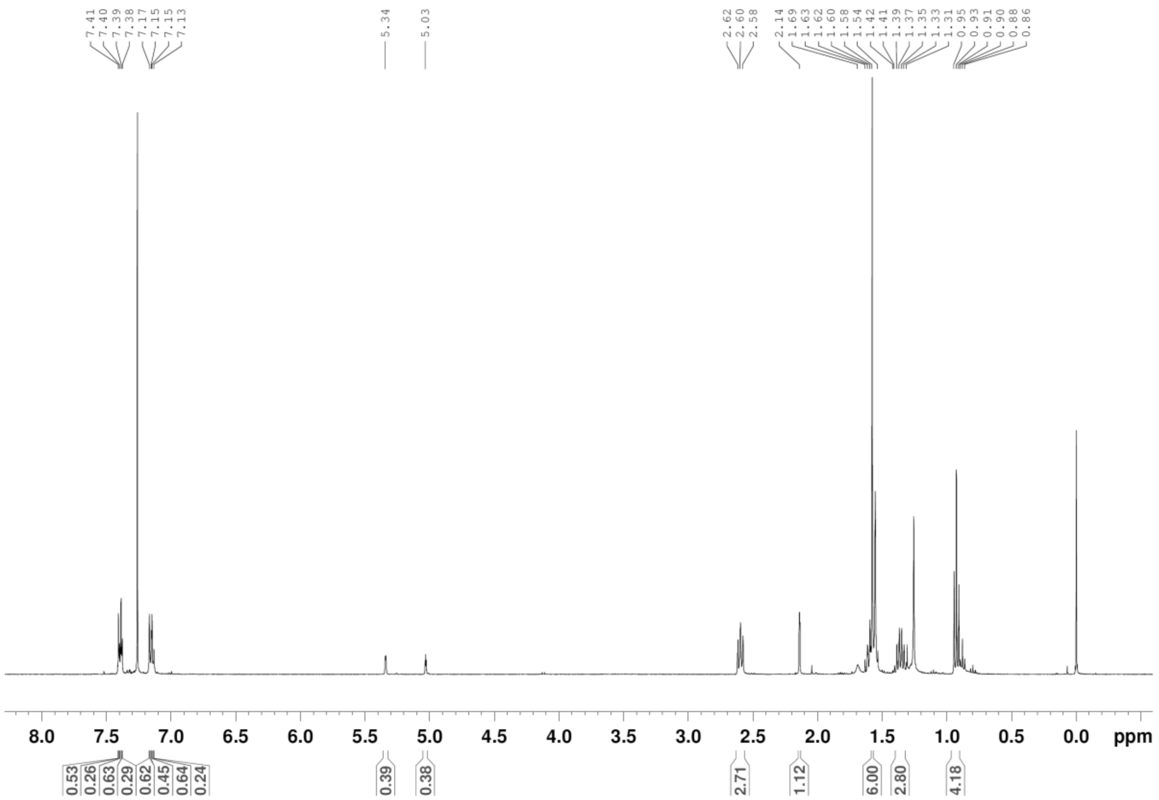

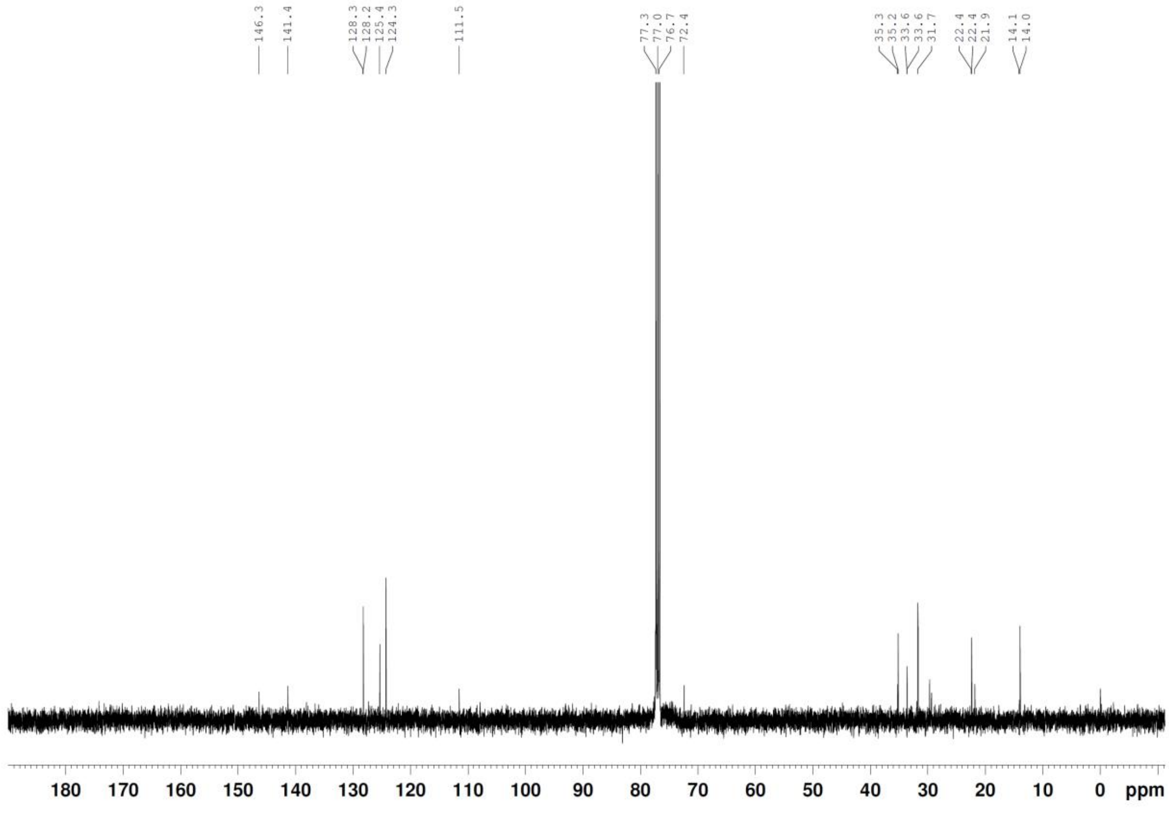

NMR-spectra for $[Me_2Al(\mu\text{-O-adamantyl})]_2$.

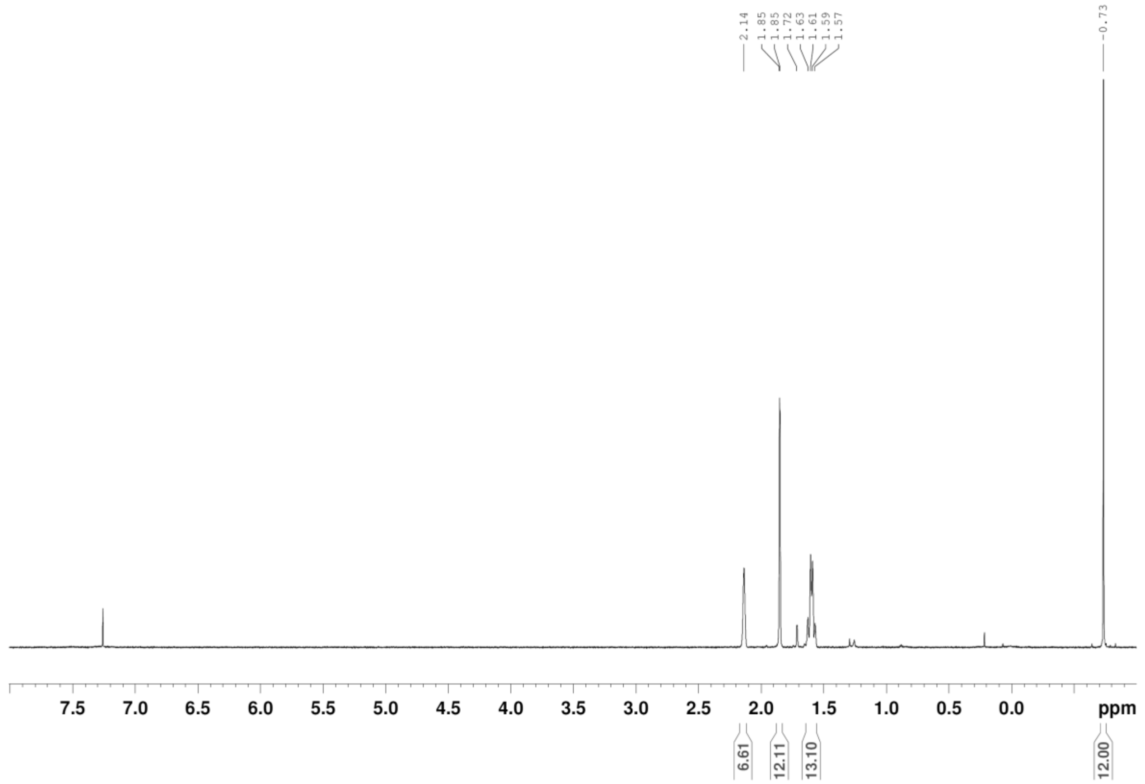

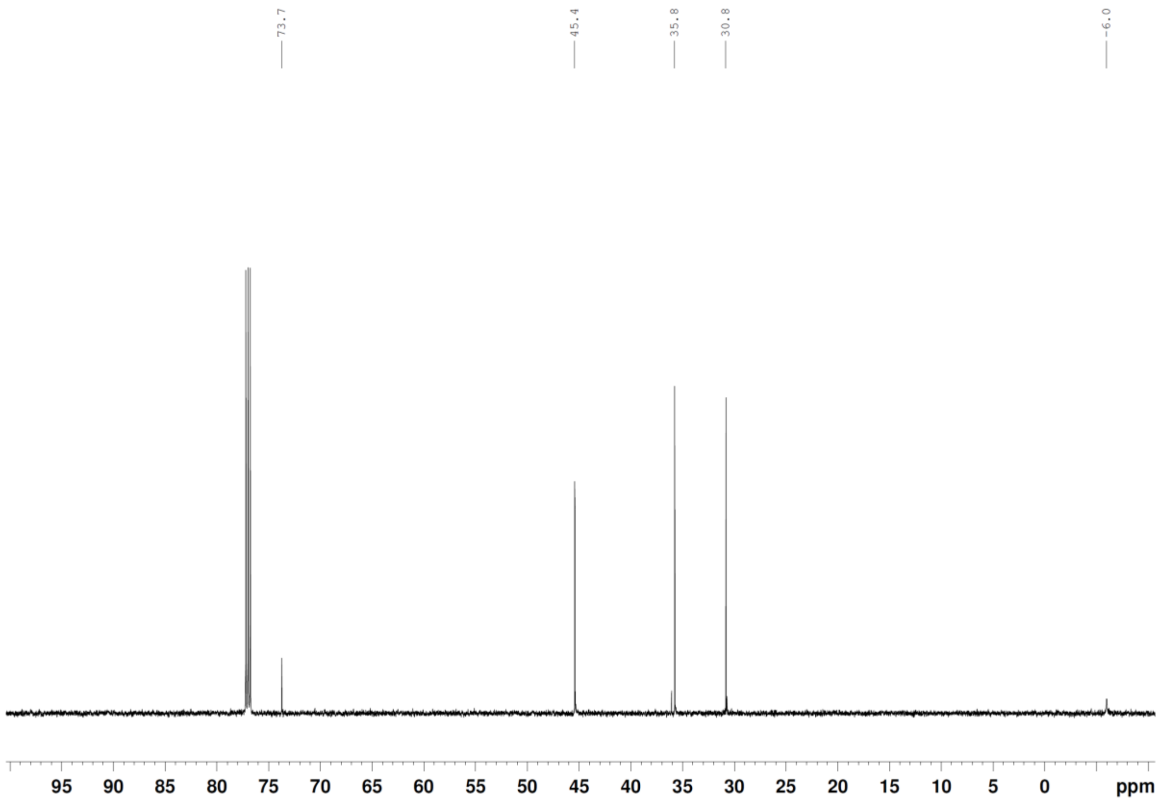

NMR-spectra for [Me_2Al(μ-O-cedrane)]$_2$.

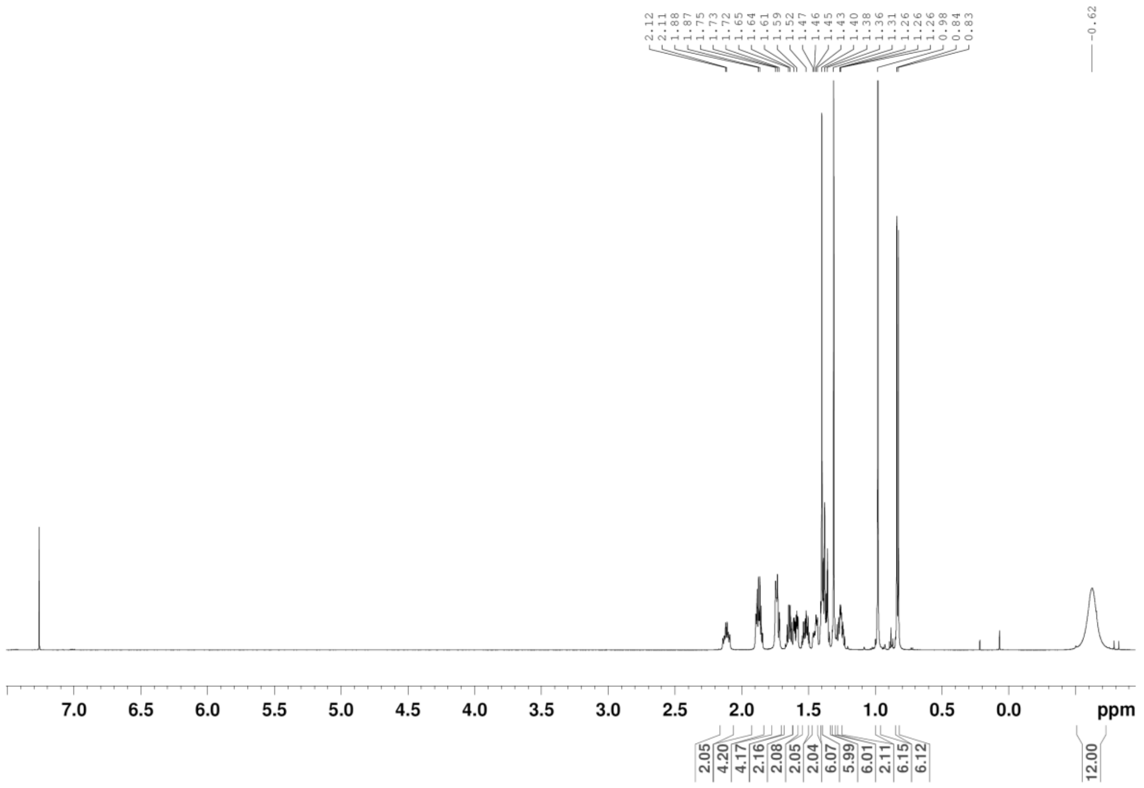

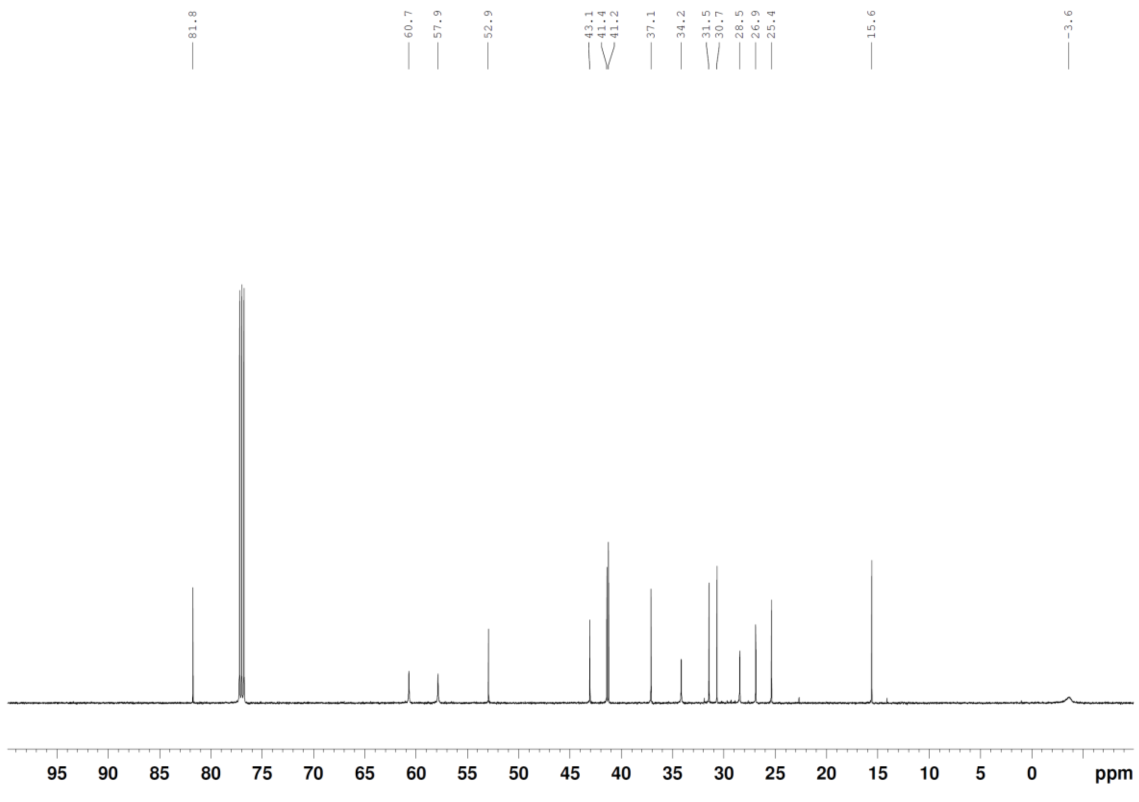

NMR-spectra for $[Me_2Al(\mu\text{-}O\text{-}cedrane)]_2$ at 70 °C.

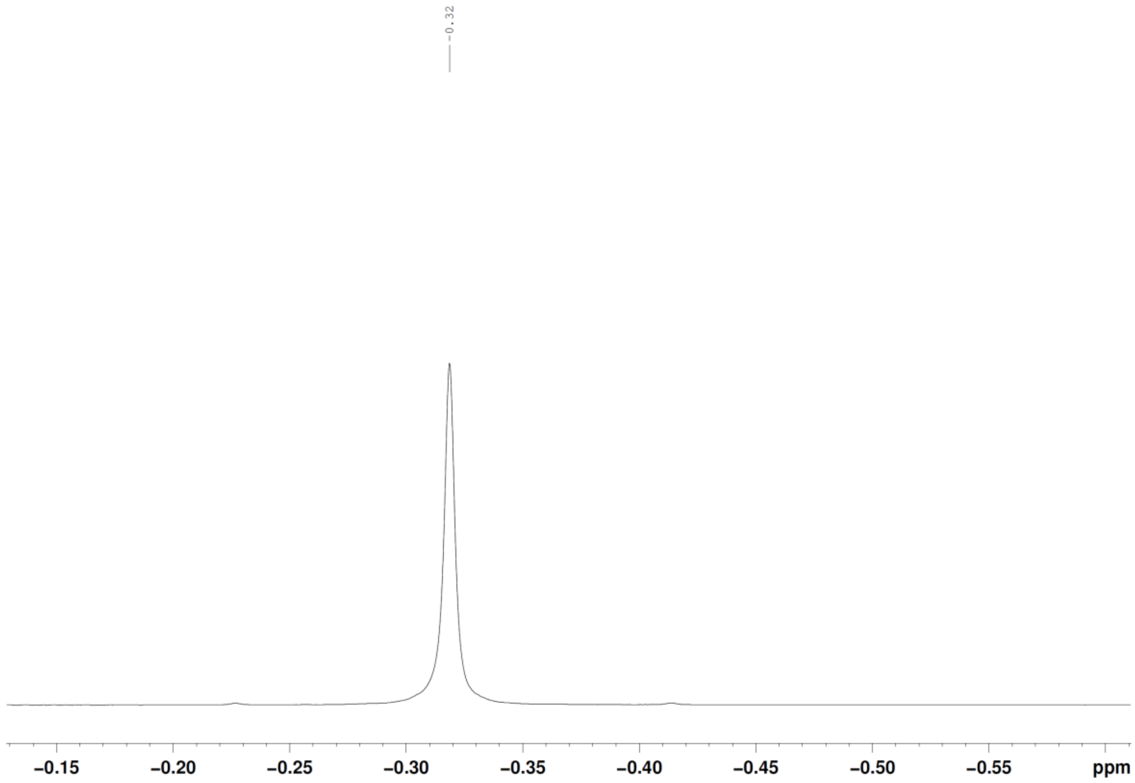

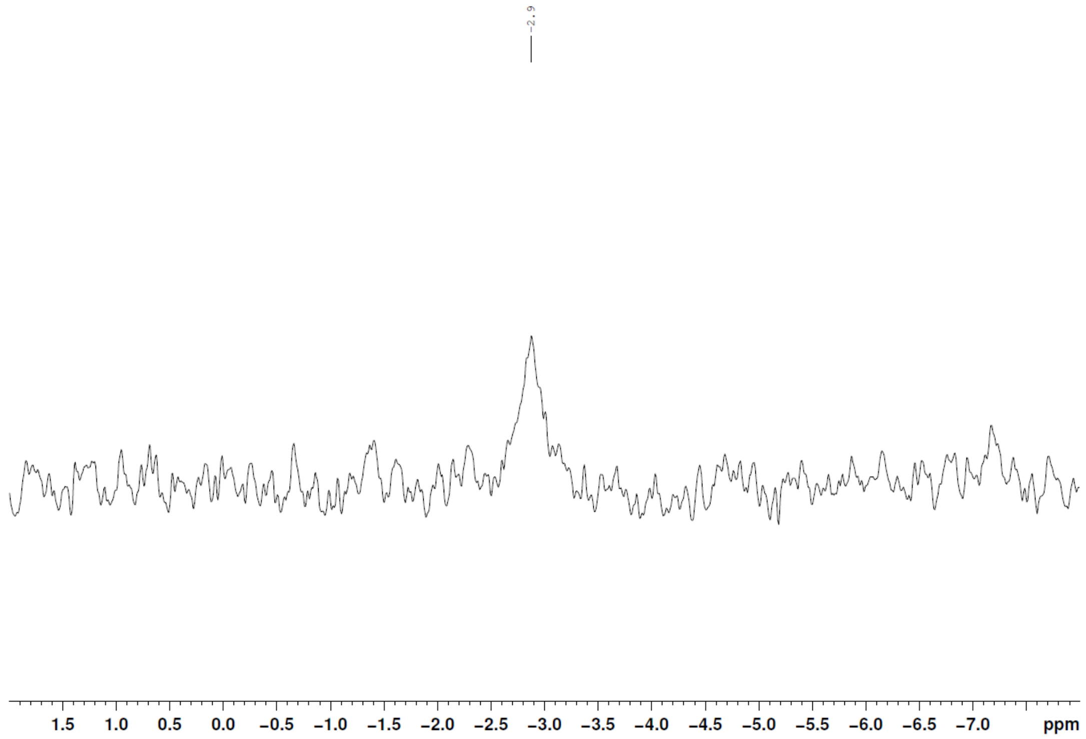

4. References

[1] Retrieved from https://github.com/ForsakenNGS/FixUp3D (4.8.2015).

[2] M. Tobisu, R. Nakamura, Y. Kita, N. Chatani, *J. Am. Chem. Soc.* **2009**, *131*, 3174.

[3] A. Meisters, T. Mole, *J. Chem. Soc., Chem. Commun*. **1972**, 595.

[4] B. Wang, H.-X. Sun, Z.-H. Sun, G.-Q. Lin, *Adv. Synth. Catal*. **2009**, *351*, 415.

[5] J. P. Pitteloud, Y. Liang, S. F. Wnuk, *Chem. Lett*. **2011**, *40*, 967.

[6] D. W. Harney, A. Meisters, T. Mole, *Aust. J. Chem.* **1974**, *27*, 1639.
